# Correlates of interpersonal emotion regulation problems in Loss of Control eating (LOC) in youth: study protocol of the combined online and App based questionnaire, laboratory and randomized controlled online intervention i-BEAT trial

**DOI:** 10.1186/s40359-021-00690-8

**Published:** 2021-12-11

**Authors:** Simone Munsch, Felicitas Forrer, Adrian Naas, Verena Mueller, Marius Rubo, Fouad Hannoun, Elena Mugellini

**Affiliations:** 1 Clinical Psychology and Psychotherapy, Department of Psychology, University of Fribourg, Rue P.-A.-de-Faucigny 2, 1700 Fribourg, Switzerland; 2 Technology for Human Well-Being Institute (HumanTech), University of Applied Sciences of Western Switzerland, Boulevard de Pérolles 80, Fribourg, Switzerland

**Keywords:** Loss of control eating (LOC), Youth, Interpersonal emotion regulation, Rejection sensitivity, Virtual reality, Cyberball task, Cross and longitudinal questionnaire study, Randomized controlled online treatment, Additive treatment effect, CBT, Emotion regulation training

## Abstract

**Background:**

Binge Eating Disorder (BED) represents a common eating disorder associated with marked health impairments. A subclinical variant, loss of control eating (LOC) is prevalent in youth. LOC is associated with similar mental distress as full-blown BED, increases the risk to develop a BED and promotes continuous weight gain. The etiology of LOC is not yet fully understood and specialized treatment for youth is scarce.

**Methods:**

The i-BEAT study includes a cross-sectional and longitudinal online questionnaire study (N = 600), an App based daily-life approach and a laboratory virtual reality study in N = 60 youths (14–24 years) with and without LOC as well as a controlled randomized online treatment trial to investigate the feasibility, acceptance and efficacy of a CBT and an interpersonal emotion regulation module for youth (N = 120). The primary outcomes include self-reported as well as measured (heart rate variability, gaze behavior, reaction times in stop signal task) associations between emotion regulation problems (such as dealing with RS), psychological impairment and binge eating in a healthy control group and youth with LOC. Secondary outcomes encompass general eating disorder pathology, social anxiety, body mass index, hyperscanning behavior and therapists’ rating of patients’ condition pre and post treatment. Epigenetic correlates of RS are assessed in healthy controls and youth with LOC and explored before and after treatment.

**Discussion:**

The expected findings will specify the role of interpersonal emotion regulation problems such as coping with the experience of social exclusion and rejection sensitivity (RS) in LOC and clarify, whether including a training to cope with RS adds to the efficacy of a cognitive behavioral treatment (CBT).

*Trial registration*: German Clinical Trial Register: DRKS00023706. Registered 27 November 2020, https://www.drks.de/drks_web/navigate.do?navigationId=trial.HTML&TRIAL_ID=DRKS00023706

**Supplementary Information:**

The online version contains supplementary material available at 10.1186/s40359-021-00690-8.

## Background

### Binge eating disorder (BED) and loss of control over eating (LOC)

Since the introduction of the fifth version of the Diagnostic and Statistical Manual of Mental Disorders (DSM-5 [1]) in 2013, BED, characterized by recurrent binge eating episodes associated with marked distress represents a separate entity in the section of feeding and eating disorders. BED is relatively common, associated with weight gain and detrimental consequences for mental and physical health [2]. Current research [3, 4] shows that BED variants are common during puberty and early adulthood, a period characterized by rapid biological changes, e.g. growth spurts, brain and secondary sex characteristics development, shifts in the accumulation of body fat. Puberty and early adulthood are furthermore accompanied by psychological challenges such as striving for autonomy and identity based on the acceptance of appearance and interpersonal competences [5]. This period at the age of c. 14 to 24 years has been named *youth* by the UN (www.unesco.org) and is critical for the onset of eating disorders (ED) [6]. A substantial group of youth experiences LOC, defined as loss of control eating over smaller food amounts which do not fulfil the criteria of an objective large food amount as required according to DSM-5, but with equivalent psychopathological relevance [7, 8]. The consequences of LOC in youth include weight gain, stress symptoms, substance use and low quality of life as well as increased suicide risks in female and male youth [9]. There is a consensus to apply age adapted criteria to capture the presentation of youthful BED variants and LOC (the two of them thereafter summarized as LOC). Existing research applies various frequency and duration criteria, from at least one LOC episode during the prior month to once a week during the previous 3 months [4, 10]. Up to now, most of the research in LOC relies on female participants, even though males are more often affected with BED and LOC than in other EDs [11].

### Epidemiology of LOC

Data on prevalence and the course of LOC in youth is scarce. According to a recent review [3] prevalence rates vary from 1 to 5% for BED and from 2.5 to 4.6% for subclinical forms of BED like LOC [12], with higher prevalence of LOC and BED in obese youth than in the general population. One of the rare longitudinal studies on the persistence of LOC in an US cohort found that LOC was significantly related to the stability of ED symptoms over the course of 10 years [13]. Another, smaller study found moderate stability of LOC over a 5-years period [14].

### Interpersonal emotion regulation as a risk factor for LOC

**Fig. 1:**
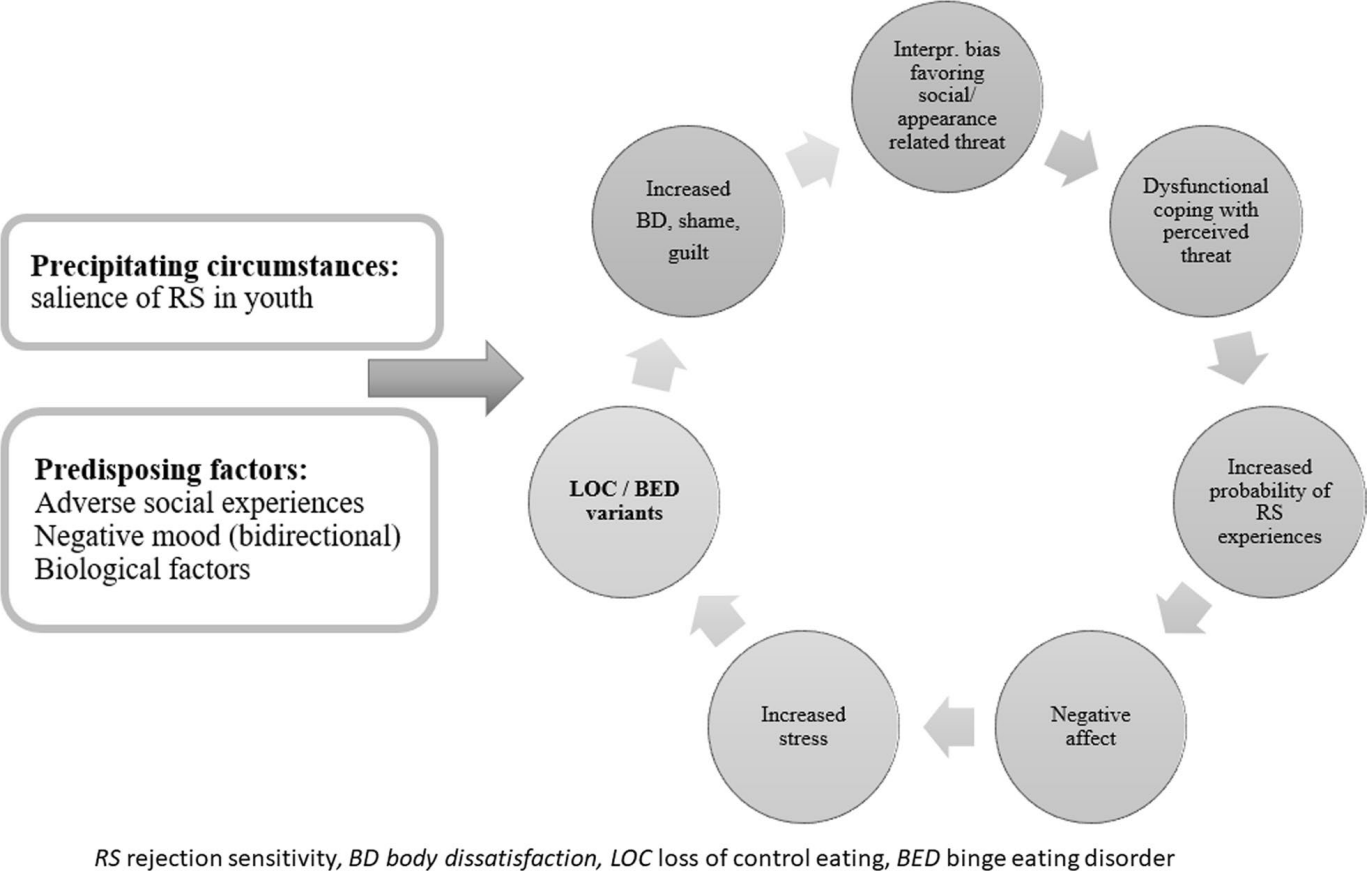
Working model of the role of interpersonal emotion regulation problems, rejection sensitivity, in youth with LOC

Recent studies on psychological risk factors for the development of disordered eating confirm *dual-pathway models*, where sociocultural influences such as an unrealistic thin ideal are assumed to lead to body dissatisfaction, which promotes dieting and binge eating (e.g. [15]). While data on the dieting pathway remains controversial, the negative affect pathway has been confirmed in cross-sectional and longitudinal studies [16–18]. Recently, the model has been applied to LOC, where dysfunctional short-term emotion regulation strategies to alleviate negative affect play an important role [18]. An impaired awareness, understanding and acceptance of emotions or few appropriate emotion regulation strategies [19] are related to interpersonal problems [20, 21]. It can be assumed that interpersonal problems in youth interact with negative affect and lead to vicious circles promoting continued LOC [22]. Research in youth with LOC has only begun to assess the role of negative interpersonal and appearance-related evaluation, which are likely to be salient at that age [23], and therefore the sensitivity to general or appearance-related rejection (referred to as rejection sensitivity, RS) also gained interest [24]. RS describes an individual’s tendency to anxiously expect, readily perceive and overact to real or imagined rejection; often associated with past and current adverse experiences [25]. In youth, questionnaire-assessed appearance-related RS independent of gender, was related to dysfunctional eating behavior, lower self-esteem and more frequent experience of appearance-related teasing [26]. So far, there is only one laboratory study in youth with LOC which underlines the effect of negative peer evaluation in a small sample of overweight to obese females. In this study, the fictitious evaluation by peers based on photographs of the participants was associated with increased activation of anxiety related brain regions and failure to engage prefrontal cortex regions involved in emotion regulation attempts, all of which might be alleviated by overeating [27]. An increased RS in interaction with impaired emotion regulation and negative affect or mood has the potential to promote the development and maintenance of LOC in youth, but needs further clarification (Fig. 1). In addition, it can be assumed that the continued experience of rejection threatens the homeostasis of a person’s milieu, leads to a prolonged physiological stress response and might contribute to the intake of palatable food at an increased eating rate [28] and therefore to overeating and LOC. The capacity of the organism to adequately respond to stress conditions depends on the flexibility of the autonomic nervous system (ANS) which is connected to the amygdala and the medial prefrontal cortex and is indexed by heart rate variability (HRV) [29]. Reduced emotion regulation abilities are common in adults with EDs, but little is known about similar difficulties in youth with LOC [30, 31]. Consequently, investigating HRV responses to being exposed to social exclusion might help to understand emotion regulation skills in stressful situations and clarify the impact on LOC.

### Treatment research in LOC

Despite the relatively high prevalence of LOC in youth and the negative effects on mental and physical health, studies on psychological treatments in youth are scarce [32]. The existing evidence from diverse samples regarding LOC in youth point to the efficacy of cognitive behavioral therapy (CBT) [33], interpersonal therapy (IPT) [34] or dialectic behavioral therapy (DBT) [35] in reducing binge eating frequency. One online treatment study applied CBT to a sample of 105 male and female high school students at risk for overweight and/ or with overeating or LOC at a mean age of 15 years. The online treatment resulted in decreased LOC episodes and a moderate weight reduction up to the 9 months follow-up [36]. Consequently, it can be assumed, that an explicit focus on eating behavior as in CBT for eating disorders (CBT-E) might be appropriate for youth [33], but there is further promise in targeting interpersonal emotion regulation deficits, leading to negative affect and LOC [34], while the differential or additive efficacy of CBT-E and interventions on interpersonal emotion regulation in female and male youth needs further investigation.

## Methods/design

### Study aims and hypotheses

The general aim of the i-BEAT (online Binge Eating for Adolescent Treatment) study is to explore the role of interpersonal emotion regulation in terms of social exclusion and RS in youth with LOC compared to a healthy control group (HCG). The investigation includes a cross-sectional and longitudinal questionnaire and an App based daily-life self-report assessment about the association of negative affect, social rejection, RS, emotion regulation difficulties and LOC episodes (study 1), a laboratory based virtual reality (VR) cyber ball task to address the effects of social exclusion on psychological and physiological correlates of stress and a gamified Stop Signal Task (gSST) to assess behavioral motor inhibition capacities (study 2) and a randomized controlled online treatment trial to investigate the separate (superiority for specific outcomes) and additive effect of a self-help CBT-E and an interpersonal emotion regulation module for eating disorders (INTER-E) (study 3).

The following hypotheses are addressed in the three substudies:

Hypotheses concerning (1) primary and (2) secondary outcomes in study 1:

1. Questionnaire and App based daily-life assessed RS at baseline is positively associated with the number of weekly LOC episodes, ED pathology, past and current exclusion experiences, emotion regulation difficulties, impaired mental health and with Body Mass Index (BMI) in all groups. Questionnaire and App based daily-life assessed RS at baseline is positively associated with the number of weekly LOC episodes, ED pathology, past and current exclusion experiences, emotion regulation difficulties, impaired mental health and with Body Mass Index (BMI) in all groups.
  Rejection sensitivity at baseline is positively associated with the number and severity of LOC episodes, with problems of emotion regulation, impaired mental health and with ED pathology at year 2.
  Emotional eating and/or LOC episodes in daily-life result in a short-term reduction of negative affect but contribute to increased body dissatisfaction, especially in youth with LOC.
2. Lower inhibition capacities precede and predict weekly LOC episodes, emotional eating and urge to eat.
  Lower inhibition capacities correlate with impaired mental health.
  Effects become stronger with increasing values of RS.

Hypotheses concerning (1) primary and (2) secondary outcomes in study 2:

1. Exposure to the exclusion condition of the VR Cyberball task leads to greater social threat-related effects during and after the exclusion (lower HRV; increased negative/reduced positive affect, impairment of basic needs) and to a delayed recovery in youth with LOC compared to the HCG. Exposure to the exclusion condition of the VR Cyberball task leads to greater social threat-related effects during and after the exclusion (lower HRV; increased negative/reduced positive affect, impairment of basic needs) and to a delayed recovery in youth with LOC compared to the HCG.
  Exposure to the exclusion condition leads to higher values of body dissatisfaction and of urge to engage in disinhibited eating after the task and during recovery in youth with LOC compared to HCG.
2. RS interpretation bias (underestimation of ball tosses while included, misinterpretation as being excluded during inclusion condition, negative evaluation of ambiguous social scenarios) is more pronounced in youth with LOC than in the HCG.
  Exposure to the exclusion condition leads to stronger pupillary dilation response, stronger hyperscanning (eye gaze), more closed hand posture, stronger aversion of social gaze (measured as gaze dwell time) in youth with LOC compared to the HCG.
  Youth with LOC show lower motor inhibition capacities indicated by longer SSRTs than the HCG during the gSST.
  The motor inhibition capacity group difference is especially apparent in the food stimuli condition of the gSST in the LOC group compared to the HCG.

Hypotheses concerning (1) primary and (2) secondary outcomes in study 3:

1. Applying an INTER-E or a CBT-E module leads to a greater reduction of the number and severity of LOC episodes, problems of emotion regulation and negative mood than a 4-weeks’ waiting period.
  The INTER-E module reduces weekly RS experiences, negative mood and problems of emotion regulation more strongly than the CBT-E module, whereas CBT-E decreases numbers and severity of weekly LOC episodes and urge to engage in disinhibited eating more importantly than the INTER-E module.
  Applying the additional CBT-E or INTER-E module results in additional improvement of RS experiences, numbers or severity of weekly LOC episodes, problems of emotion regulation and urge to engage in disinhibited eating.
2. BMI remains stable during active treatment and follow-up.
  Effects of the initial INTER-E or the CBT-E module remain stable during a 3-weeks pause.
  Effects of the treatment remain stable during 6- and 12-months follow-up.
  Therapist ratings of improvement are in line with the self-report of the patients.
  CBT-E and INTER-E result in similar acceptance, usability, attrition and adherence rates.

We further aim to explore epigenetic underpinnings (DNA methylation, DNAm) of RS and emotion regulation capacity [37]. DNAm patterns are thought to reflect exposure to various psychosocial influences across the life-span and have been shown to relate to behavioral, emotional and cognitive styles especially in youth [38, 39]. We seek to explore the DNAm levels of genes involved in stress regulation and social interaction (FK-506-binding protein 5 (FKBP5) [40], the glucocorticoid receptor (NR3C1) [41], oxytocin receptor (OXTR) [42, 43] and the serotonin transporter (SLC6A4) in youth with LOC compared to a HCG (prior to treatment) and in LOC prior versus post treatment.

### Study design

This study protocol has been written according to the SPIRIT statement (Standard Protocol Items for Randomized Trials) [44].

**Fig. 2:**
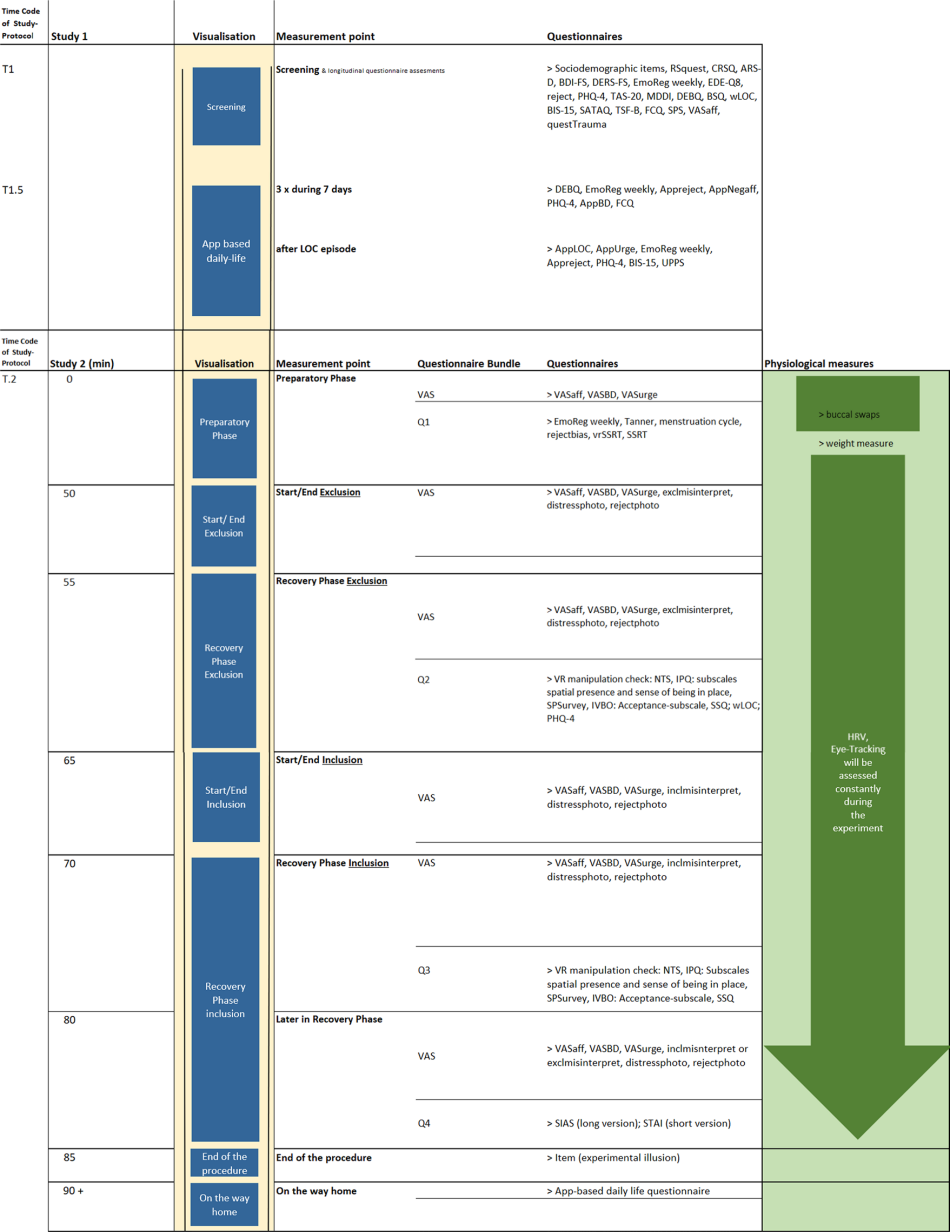

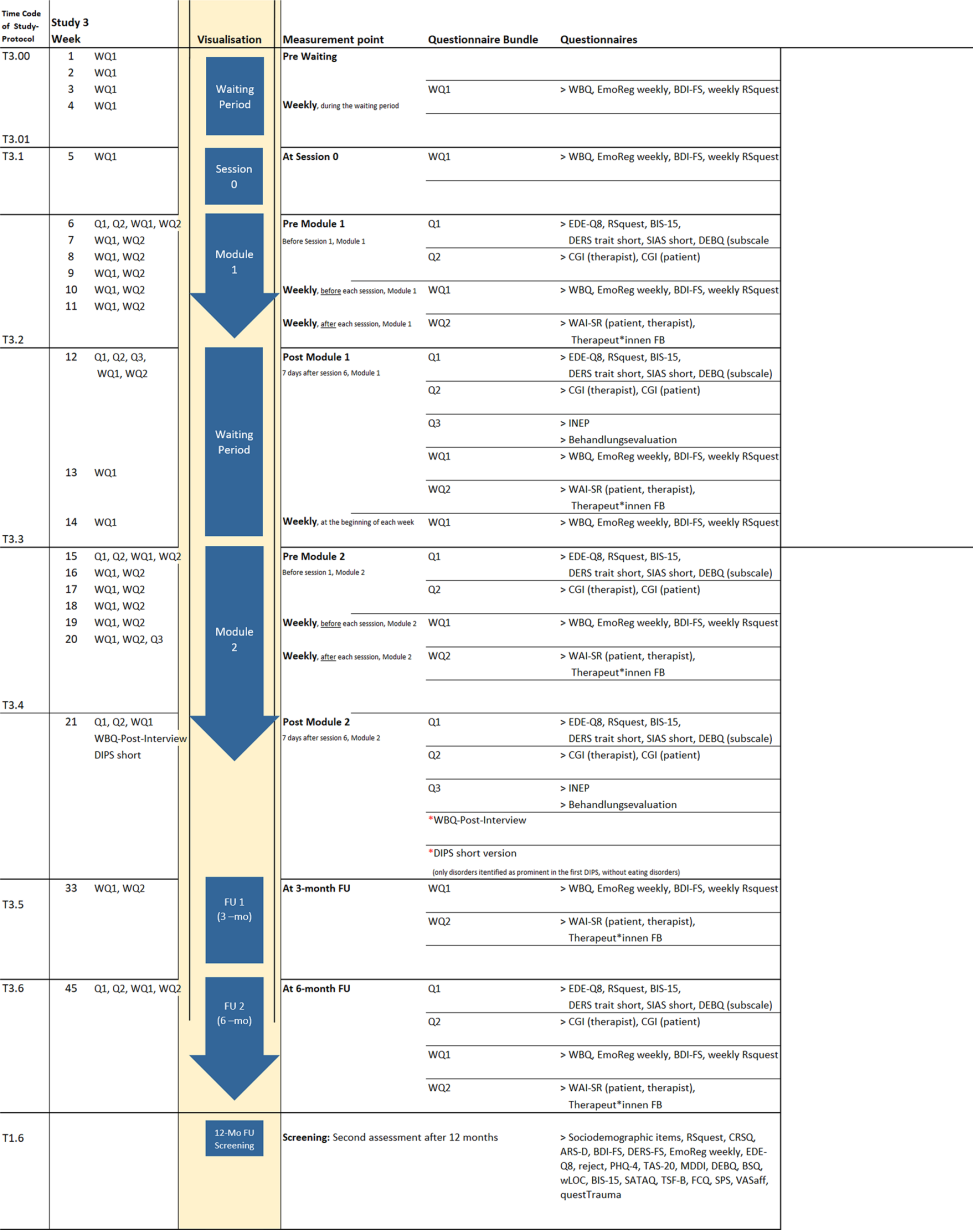
Instruments. *Note.* (1) The complete DIPS will be conducted after the questionnaire-based screening study 1 and before further participation in either the laboratory study 2 or the online treatment study 3. The DIPS will only be conducted, if participants indicated their interest in either study 2 or study 3. (2) The Weekly Binges Post-Interview (WBQ-Post-Interview) is performed at the measurement point "Post Module 2", see above. (3) The DIPS short version will be conducted again at measurement point "Post Module 2". This short version only checks for disorders that have been identified as prominent in the first DIPS, and does not ask for eating disorder pathology. Note, almost all questionnaires and scales that will be used in i-BEAT study have been validated in German in the past. The respective citations are presented in the list of abbreviations. Only few questionnaires have been developed for the project at hand and are marked as “self-developed”. For a more detailed overview of the instruments, see additional file 1, "Instruments"

The i-BEAT project includes a multimethod three study arms approach. Study 1 encompasses a cross sectional and a longitudinal questionnaire-based survey. At baseline and one year later, questionnaire-based data is acquired online and the sample is screened to identify youth with LOC and HCG. The procedure is based on our pilot study BEAT (Binge Eating for Adolescent Treatment; DRKS00014580). App based daily-life data is assessed in a subgroup with LOC and in a HCG. We compare changes concerning the primary and secondary end points of study 1 between LOC versus HCG at T1.0, after one year at T1.6 and repeatedly during the 7-days of the App based daily life study (T1.5). Study 2 applies an adapted VR Cyberball task in the laboratory [45] and primary and secondary outcomes are compared between youth with LOC and age and gender matched HCG youth. Study 3 is a randomized wait-list between and within-subject online intervention study with a repeated measures design to evaluate the separate and additive effect of each of the two 6-weeks CBT-E or INTER-E modules (within subject effect) up to 6 and 12-months follow-up. After completion of the first module, participants wait 3 weeks until they receive the other module. We compare changes concerning the primary and secondary end points in participants from T3.00 to T3.1, T3.1 to T3.2 (active treatment in module 1), T3.2 to T3.3 (three weeks waiting between modules) and T3.3 to T3.4 (active treatment in module 2) as well as from T3.4 to the follow ups T3.4 and T3.5 (see Fig. 2 for study procedure).

### Participants

**Fig. 3:**
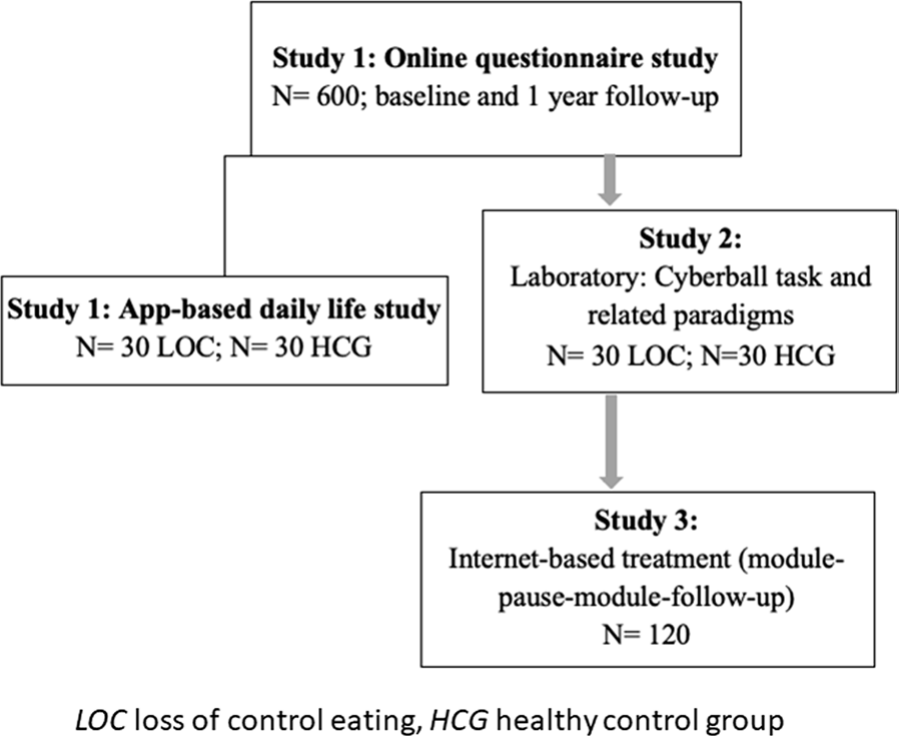
Study sample

Study 1 serves as a tool to increase awareness and recruit youth suffering from LOC and a HCG participating in all substudies. Youth with LOC participating in study 3 are randomized according to a permuted block design [46] to CBT-E or INTER-E first. Participants’ inclusion criteria for studies 1–3 are age between 14 and 24 years, sufficient German language competences and written informed consent. Criteria for LOC [47] are fulfilled and youth included if they experience at least 3 episodes of LOC during the last 3 months accompanied by at least 3 out of 5 behavioral indicators and/or some degree of distress, absence of AN or BN. Inclusion criteria for youth participating in the HCG are healthy body weight (BMI 18.5–24.9), absence of any past or present ED and absence of any present mental disorder according to the diagnostic interview. Youth from the LOC group are excluded if they suffer from any current mental disorder preventing safe participation in the i-BEAT study or if there is an intake of weight affecting drugs, participation in an ED related psychotherapy or weight loss treatment. Females in pregnancy or lactation are excluded. Additional in- and exclusion criteria for study 2 are intact or corrected vision capacity and nausea in VR. Figure 3 illustrates the study sample.

### Sample size

For study 1 we will recruit N = 600 youth and expect a participation rate of c. 70–80% after one year. Power is sufficient (1−β = 0.8) to detect small to medium effect sizes *f*^2^ of c. 0.08, applying multiple regression models including covariates and testing specific predictors, for a given β of 0.05. For the App based study 1 multilevel models are applied to carry out between-subjects analysis of covariance. Moderators will be included to test for the interaction effects between groups (LOC vs HCG) within the Cyberball task. We expect moderate to large effect sizes (d = 0.8) with sufficient power to detect significant effects given 1−b = 0.8 and a = 0.05 for study 2 with N = 60 youths, even when accounting for c. 13% dropouts. For study 3, mixed between and within-subjects analysis of covariance or linear mixed models are carried out. Based on our BEAT pilot trial (DRKS00014580), where a high effect size for within subject measures revealed (d = 1.37) a medium to high effect size for these effects can be assumed (f = 0.25, taking r = 0.5 for the correlation among repeated measures), the required sample size would be N = 34 with sufficient power to detect significant effects given 1−β = 0.8 and β = 0.05 for study 3 with N = 120 youths, even when accounting for dropouts. Exploratory epigenetic goals involve predictors and within-subject effects and are expected to be sufficiently powered if effect sizes are medium to large (Fig. 3 depicts the study sample).

### Recruitment

Recruitment efforts include media, posting on websites, contacting health care institutions and secondary, professional schools or Universities in the German speaking part of Switzerland [33]. In case of positive LOC scores or interest in study participation as part of the HCG, an online screening will be conducted and eligibility, diagnostic status, in and exclusion criteria will be verified (see Fig. 2). The first participant in study 1 is planned to be enrolled in March 2021.

### Procedure and interventions

#### Cyberball task in virtual reality (VR)

The procedure of the i-BEAT study is illustrated in Fig. 2. Each participant is eligible to take part at one, two or at each of the three studies.

Interested youth are provided with a link to participate in study 1 and if eligible invited to participate in the App based study 1, study 2 and/or 3. Study 2 takes place between 2 and 4 PM after a regular meal at home. Upon arrival at the VR Lab at the Department of Psychology, University of Fribourg, First, electrodes to measure HRV (biosignalsplux, https://plux.info/12-biosignalsplux) are placed and a photograph of the participants is taken. HRV will be assessed continuously during the whole experiment.

With respect to eye tracking, we will use an HTC Vive Pro Eye (2160 × 1200 pixel resolution, 110° field of view) with a built-in eye-tracker (Tobii) to display the VR scene and concurrently collect gaze direction data at a sampling rate of 60 Hz. Gaze rays in 3D space obtained for both eyes will be geometrically transposed to represent horizontal and vertical deviations from regions of interest (as introduced in [48]). The procedure results in a form of data representation which is comparable to that obtained in traditional stationary eye-tracking where gaze deviations are described along a screen’s x- and y-axes. This form of data representation allows to apply a drift correction if samples directed towards regions of interest exhibit a drift over time (e.g. caused by a gradual lowering of the HMD on the participant’s head). After removing data obtained during blinks, data from both eyes will be averaged. Gaze will be labeled as either resting on a conspecific’s face (if deviation to any of the two faces present in the scene is lower than two degrees visual angle) or elsewhere.

**Fig. 4:**
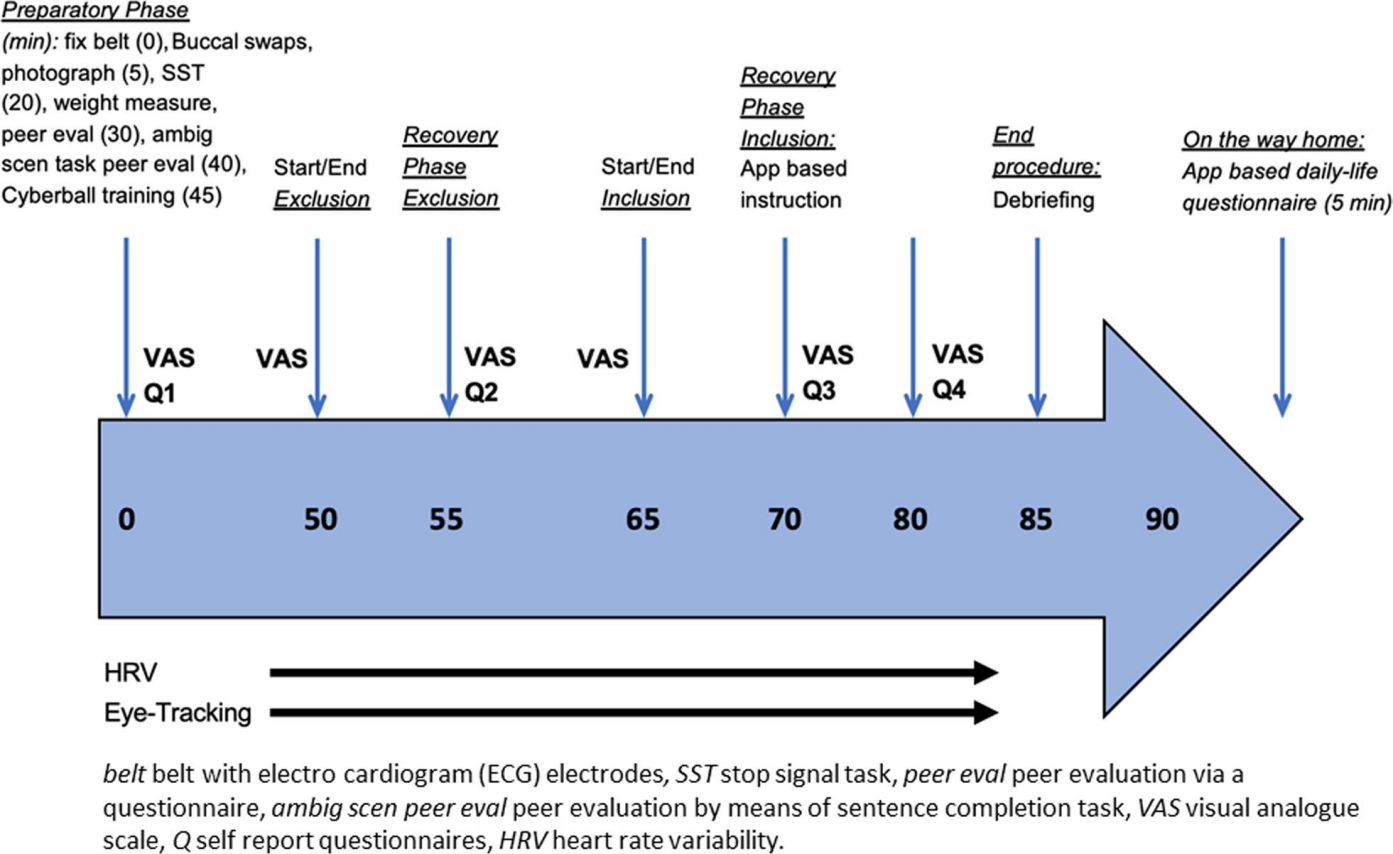
Experimental procedure during Cyberball-task in virtual reality, VR

At the beginning of the preparatory phase (see Fig. 4), participants self-report their psychological well-being and complete questionnaires (see Fig. 2). They are introduced to the adapted Cyberball task [27] (developed using the Unity 3D Game Engine) based on our pilot BEAT study and are informed that they will play with 2 peers and are shown pictures of these peers. Then, the motor-inhibition task, the gSST is applied. After a training trial with the oculus rift VR headset and oculus touch controller (preparation phase), the Cyberball task starts. All participants are exposed to the exclusion condition first followed by the inclusion condition. The order of the conditions is kept secret. The psychological effects of experiencing exclusion are repeatedly assessed during the VR Cyberball task. Participants rate how excluded or ignored they felt (manipulation check) as well as the percentage of received ball tosses. During recovery phase (see Fig. 4), they receive instructions in the App based daily-life study and during the preparatory phase, buccal cell samples to measure DNAm of candidate genes are provided (see Fig. 2). DNA methylation will be assessed using targeted bisulfite sequencing. In brief, bisulfite treated DNA will undergo two rounds of PCR (1st round: amplification for specific target regions; 2nd round: introduce identifiers for individuals), and will be sequenced on a MiSeq platform. To capture possible medium-term effects as a result of ostracism, a short questionnaire will be filled-out by all participants on their way home via the daily-life App and participants are debriefed about the purpose of study 2.

#### Online self-help treatment for LOC

Based on our previous work in BED in adults [49, 50] and based on our preliminary results of the blended treatment in our pilot BEAT study (active treatment consisting of 3 face to face sessions and 6 weekly e-mail guided self-help sessions, followed by 1, 3, 6, and 12-month follow-up sessions), we developed an online self-help treatment consisting of two distinct modules CBT-E and INTER-E (see Table 1). To increase compliance and decrease dropouts, therapists provide guidance via an Email generated system within the online platform [51]. Therapists (psychologists attending a post graduate training in CBT psychotherapy) will be trained and regularly supervised by SM.

**Table 1:** Content of CBT-E and INTER-E treatment modules

0. Motivation & Psychoeducation: Short (20 min) introduction and welcome session of the i-BEAT treatment trial	
Advocatus Diaboli	
Introduction of main aspects (e.g., binge eating, emotion regulation)	
Introduction of the protagonists	
Explanation of the i-BEAT online platform	
Cognitive behavioral treatment (CBT-E)	Interpersonal emotion regulation (INTER-E)
1. Motivation & Psychoeducation	1. Motivation & Psychoeducation
Psychoeducation: What is a healthy, balanced diet?	Psychoeducation: emotion regulation, rejection sensitivity
Introduction of the meal plan	Introduction of the interpersonal emotion regulation model
Introduction of self-observation-protocols	Self-report of social rejection and rejection sensitivity episodes
2. Personal goal setting and ABC model	2. Relaxation & Non-judgmental perception
Goal analysis and goal attainment scale	Personal vicious circle of negative emotions
Introduction of the ABC model of binge eating episodes (video)	Introduction of different strategies to overcome this vicious circle
	Progressive muscle relaxation (video)
	Non-judgmental perception of negative emotions
3. The vicious circle of binge eating & PPP	3. Acceptance & Emotional tolerance
Individual vicious circle model	Personal vicious circle of negative emotions
predisposing factors, precipitating factors and	Introduction of different strategies to overcome this vicious circle
perpetuating factors	Acceptance of negative emotions
	Tolerance of negative emotions (video)
4. Craving & Strategies	4. Analysis of triggers & Maintenance factors
The craving curve	Self-report and analysis of negative emotions, interpersonal conflicts and rejection sensitivity
Symptom management: introduction of stimuli and	Identification of risk situations
reaction control strategies; create and apply	
corresponding emergency cards	
5. Cognitions	5. Effective self-support in interpersonal conflicts
Introduction concept dysfunctional thoughts	Role of emotion regulation in interpersonal conflict situations (audio)
Strategies to cope with dysfunctional thoughts:	Coping with negative emotions, interpersonal conflicts and rejection sensitivity
Defusion (audio)	
accept difficulties as part of behavioral change	
6. Setting up and relapse prevention:	6. Coping with negative emotions, interpersonal conflicts & rejection sensitivity
Identify high-risk situations, plan coping strategies,	Evaluation of self-observations with respect to emotion regulation and rejection sensitivity
adapt emergency cards	Analysis of experiences with emotion regulation strategies
score goal attainment on scale, adapt goal or set	Identifying a top 3 of the therapy content
new goals	
Identifying a top 3 of the therapy content	
13./14.: 3–month follow-up & 6-month follow-up booster sessions	
Repetition of core elements of both treatment trials: CBT-E and INTER-E	
15./16.: 2 optional 2 CBT-E sessions assigned in individual cases with more pronounced compensatory behaviour after both i-BEAT modules	

*CBT-E* cognitive behavioural therapy for eating disorders, *INTER-E* interpersonal psychotherapy for eating disorders

### Outcome measures

Data assessors are trained and supervised in diagnostics, application of experimental procedures of study 1–2. The time schedule of the data assessment during i-BEAT studies is presented in Fig. 2.

### Diagnostic assessment

Body weight and height are self-reported in study 1 and measured upon arrival for study 2 in light clothing to compute BMI (kg/m^2^). The diagnostic interview according to DSM-5, "Diagnostisches Interview bei Psychischen Störungen" (DIPS; [52]) is applied to identify mental disorders and LOC criteria, which are further confirmed via the Eating Disorder Examination Questionnaire (EDE-Q, German version; [53]). Frequency and severity of LOC or binge eating episodes are measured using the self-constructed weekly binge eating questionnaire (WBQ; [50, 54]). Measures and assessment time points during the i-BEAT study are presented in Fig. 2. In study 3, besides pre to post and follow-up measurements, the treatment process is assessed by weekly online questionnaires provided via the platform encompassing the primary outcomes frequency and severity of weekly LOC episodes, weekly interpersonal emotion regulation problems/ RS as well as attrition, adherence, dropouts, acceptance and feasibility.

### Data analysis

Data will be analyzed with the Statistical Package of Social Sciences (SPSS) and R. We will apply multiple regression models including covariates and testing specific predictors (study 1), multilevel models including moderators (App based study 1), between subjects analysis of covariance including moderators (study 2) and mixed between and within subjects analysis of covariance or linear mixed models (study 3). Exploratory epigenetic goals involve predictors and within-subject effects. Expected dropouts are assumed to amount up to 10–18%. Random occurrence should lead to equal distribution of dropouts in the different groups (LOC vs. HCG; CBT-E first vs. INTER-E first). However, in the case of an unequal distribution of the number of participants to the different groups, this issue will be addressed with multi-level models. Multi-level models are assumed to be robust against violations of equal group sizes [55]. Additionally, we will test whether a missing at random pattern is a reasonable assumption with respect to dropout.

## Discussion

The overarching goal of this project is to better understand the role of interpersonal emotion regulation problems, especially when feeling rejected (RS), in youth suffering from LOC. LOC importantly promotes EDs, general psychopathology and interferes with the development of a positive identity and the capability to engage in meaningful relationships. The interventional part of the project will show how youth accepts, reacts to and benefits from a CBT-E and an INTER-E module and whether there are specific and additive effects. The i-BEAT study represents a multi-method approach including psychological, behavioral, psychophysiological and epigenetic data, which is unique in the field and generates interdisciplinary evidence.

### List of abbreviations and questionnaire sources

- Adapted German version of the Sociocultural Attitudes Towards Appearance Questionnaire, [56].
- Adapted German version of the Thought Shape Fusion questionnaire (TSF-B; [57]).
- Adapted Rejection Sensitivity Questionnaire (RSquest, adapted from the RSQ, [58]; CRSQ, German version, FZE-K, [59]).
- Adapted version of the Brief Mood Scale, Three-dimensional Affect Scale, [61].
- Adapted Working Alliance Inventory (WAI-SR; [62]).
- Ambiguous Scenario-Sentence Completion Task; Cognitive Bias Modification-Interpretation (rejectbias; according to [63]).
- App-based adapted Three Dimensions Affect scale (AppNegaff; [61, 64]).
- App-based adapted Visual Analog Scale Body Image Satisfaction (AppBD; [64]).
- Rejection Sensitivity Qu [60]).
- Autonomic Nervous System (ANS).
- Barratt Impulsiveness Scale (BIS-15; [65]).
- Beck Depression Inventory Fast Screen German (BDI-FS; [66]).
- Binge Eating Adolescents and young adults Training (i-BEAT).
- Binge Eating Disorder (BED).
- Body Mass Index (BMI).
- Body Shape Questionnaire (BSQ; [67]).
- Brief Mood Scale (BMS; [64]).
- CBT for eating disorders (CBT-E).
- .
- Clinical Global Impression scale (CGI; [68]).
- Cognitive Behavioral Therapy (CBT).
- Diagnostic and Statistical Manual of Mental Disorders (DSM-5).
- Dialectic Behavioral Therapy (DBT).
- Diagnostisches Interview bei Psychischen Störungen (DIPS).
- Difficulties in Emotion Regulation Scale (DERS; [69]).
- Difficulties in Emotion Regulation Scale—Short Form (DERS-SF; [70]).
- DNA methylation (DNAm).
- Dutch Eating Behavior Questionnaire (DEBQ; German version; [71]).
- Eating Disorder Examination- Questionnaire Kurzversion (EDE-Q8; [53]).
- Eating Disorder(s) ED(s).
- EmoReg weekly - Self-developed and adapted from the DERS ([69]), the TAS-20 [72], the H-FERS [73] and the TOMS [74].
- Final evaluation of the online-therapy; self-developed items (Therapeut*innen Fragebogen).
- Food Craving Questionnaire (FCQ; [75]).
- Gamified Stop Signal Task (gSST).
- Healthy Control Group (HCG).
- Heart Rate Variability (HRV).
- Heidelberger Fragebogen zur Erfassung von Emotionsregulationsstrategien (H-FERS; [73]).
- Igroup Presence Questionnaire (IPQ; [76]).
- Illusion of Virtual Body Ownership Scale (IVBO; [77]).
- Inventar zur Erfassung negativer Effekte von Psychotherpie (INEP; [78]).
- Interpersonal Emotion Regulation module for eating disorders (INTER-E).
- Inter-Personal Therapy (IPT).
- Loss Of Control eating (LOC).
- Modified version of the Weekly Binges Questionnaire (wLOC, AppLOC, AppUrge, WBQ; [79]).
- Need Threat Scale (NTS; [80]).
- Patient Health Questionnaire for Depression and Anxiety (PHQ-4; [81]).
- Perception of Teasing Scale (POTS; [82]).
- Questionnaire on frequency and intensity of rejection experiences and bullying (reject; adapted from [83, 84]).
- RS interpretation bias: Visual Analogue Scale of misinterpretation of being excluded in the Cyberball game (exclmisinterpret; [85]).
  Rejection sensitivity, RS.
- RS interpretation bias: Visual Analogue Scale of misinterpretation of being included in the Cyberball game (inclmisinterpret; [85]).
- Short version of the Social Phobia Scale (SPS; [86]).
- Simulator Sickness Questionniare (SSQ; [87]).
- Social Interaction Anxiety Scale (SIAS; [86]).
- Sociodemographic items (self-developed items).
- Standard Protocol Items for Randomized Trials (SPIRIT).
- State-Trait Anxiety Inventory, short-version (STAI; [88]).
- Stop Signal Task, (vrSSRT, SSRT; [89]).
- Tanner questionnaire (Tanner; [90]).
- Tolerance of Mood States Scale (TOMS; [74]).
- Translated and adapted Social Presence Survey (SPSurvey; [91]).
- UPPS Impulsive Behaviour Scale (UPPS; [92]).
- Virtual Reality (VR).
- Visual Analog Scale Body Image Satisfaction (VASBD; [64]).
- Visual Analog Scale - distress photo (distressphoto; self-developed items).
- Visual Analog Scale - rejection (rejectphoto; self-developed items).
- Visual Analog Scale - urge to eat (VASurge; self-developed items based on [93]).
- Weekly Binges Questionnaire (WBQ; [79]).

## Supplementary Information

**Additional file 1**. Instruments.

## Data Availability

Youth will give informed consent to the open data access according to the Swiss National Foundation Foundation (http://www.snf/SiteCollectionDocuments/FAIR data repositories examples.pdf). The datasets generated and/or analyzed during the current study will be available in ZENODO (https://zenodo.org).
