# Supplementary material for: Correlates of interpersonal emotion regulation problems in Loss of Control eating (LOC) in youth: study protocol of the combined online and App based questionnaire, laboratory and randomized controlled online intervention i-BEAT trial
Source: BMC Psychol. 2021 Dec 11;9:193. doi: 10.1186/s40359-021-00690-8 (PMC8666071; doi:10.1186/s40359-021-00690-8)
Supplement: Supplementary file 1 — Additional file 1. Instruments. [file 40359_2021_690_MOESM1_ESM.pdf]

SPIRIT 2013 Checklist: Recommended items to address in a clinical trial protocol and related documents*

Section/item	Item No	Description
**Administrative information**		
Title	1	Descriptive title identifying the study design, population, interventions, and, if applicable, trial acronym<br><br>Correlates of interpersonal emotion regulation problems in Loss Of Control eating (LOC) in youth: study protocol of the combined online and App based questionnaire, laboratory and randomized controlled online intervention i-BEAT trial<br>i-BEAT: Binge-Eating Adolescent and young adults Treatment<br>*See p. 1-2 of the study protocol manuscript*
Trial registration	2a	Trial identifier and registry name. If not yet registered, name of intended registry<br>German Clinical Trial Register: DRKS00023706. Registered 27 November 2020.<br>*See p. 2 of the study protocol manuscript*
	2b	All items from the World Health Organization Trial Registration Data Set<br>Table 3, information on trial registration is available as a separate appendix. For all contents of the registered trial also refer to: https://www.drks.de/drks_web/navigate.do?navigationId=trial.HTML&TRIAL_ID=DRKS00023706
Protocol version	3	Date and version identifier<br>First version of the protocol, submitted on November 27th 2020.<br>No amendments submitted yet.
Funding	4	Sources and types of financial, material, and other support<br>Swiss National Science Foundation, SNSF, funded the project based on peer-reviewed procedure in 2019 for 4 years. The funding covers salary of PhD candidates, Post docs, material for experimental procedure, recruiting as well as compensation for participants.

Roles and responsibilities	5a	Names, affiliations, and roles of protocol contributors<br>Simone Munsch[1], Felicitas Forrer[1], Adrian Naas[1], Verena Mueller[1], Marius Rubo[1], Fouad Hannoun[2] and Elena Mugellini[2]<br>[1]Clinical Psychology and Psychotherapy, Department of Psychology, University of Fribourg, Rue P.-A.-de-Faucigny 2, Fribourg, Switzerland<br>[2]Technology for Human Well-being Institute (HumanTech), University of Applied Sciences of Western Switzerland, Bouvard de Pérolles 80, Fribourg, Switzerland<br>The Sponsor and main responsible person for the study, Simone Munsch, designed the outline and rational of all studies: cross- and longitudinal questionnaire study, App-based study, Cyberball paradigm in virtual reality (VR) and the online treatment study; Felicitas Forrer is responsible for the online treatment study and supports the conductance of the whole study and the integration of each substudies' ongoing developments and findings; Adrian Naas elaborated the submission to the ethical committee, co-developed the online game to assess impulsivity, assisted the development of the online platform and contributed to the sessions' contents of the online treatment, Verena Müller co-developed the questionnaire-based and App-based study and contributed to the sessions' contents of the online treatment. Marius Rubo elaborated the procedure of the Cyberball paradigm in VR and is responsible for associated procedures in the VR lab. Elena Mugellini and Fouad Hannoun developed the techniques of the online platform and will be responsible for ongoing supervision of technical procedures regarding the treatment study.
	5b	Name and contact information for the trial sponsor<br>Simone Munsch, simone.munsch@unifr.ch, Department of Psychology, University of Fribourg, Switzerland
	5c	Role of study sponsor and funders, if any, in study design; collection, management, analysis, and interpretation of data; writing of the report; and the decision to submit the report for publication, including whether they will have ultimate authority over any of these activities<br>Members of the i-BEAT study team are all academic collaborators and will have access to data in the frame of their academic career steps: PhD, post doc and further. All publications will be in accordance with the Sponsor, Simone Munsch, who will guarantee open access to data and publication wherever possible. Reports to clinical trial registry and ethical committee will be provided by Felicitas Forrer and revised by the Sponsor, Simone Munsch.
	5d	Composition, roles, and responsibilities of the coordinating centre, steering committee, endpoint adjudication committee, data management team, and other individuals or groups overseeing the trial, if applicable (see Item 21a for data monitoring committee)<br>At the end of the study funding period, the Sponsor, Simone Munsch will write a report to the SNSF.

Background and rationale	6a	Description of research question and justification for undertaking the trial, including summary of relevant studies (published and unpublished) examining benefits and harms for each intervention<br>Since the introduction of the DSM-5 in 2013, binge eating disorder (BED), characterized by recurrent binge episodes associated with marked distress represents a valid diagnostic entity in the section of feeding and eating disorders of the 5th version of the Diagnostic and Statistical Manual of Mental Disorders (DSM) (American Psychiatric Association, 2013). BED is associated with repeated ruminations and worries over shape, weight and eating and with overweight and obesity and has detrimental consequences for mental and physical health (Amianto, Ottone, Daga, & Fassino, 2015). BED is relatively common in the general population and prevalence rates are high in the obesity subgroup (Kessler et al., 2013). Even though BED has been named a typical adult eating disorder, there is increasing evidence that loss of control eating (LOC) over different quantities of food and with different frequencies is prevalent in adolescents and young adults (youth). LOC is associated with similar mental health impairments, increases the risk to develop a BED and promotes continuous weight gain and body dissatisfaction during the important developmental phase of youth. The etiology of BED and LOC is not yet fully understood. Especially in youth, psychological factors such as dysfunctional emotion regulation (bingeing in order to cope with adverse mood) in social situations when feeling rejected (rejection sensitivity) seems to play an important role and deserves further investigation. The impact of biological factors such as physiological correlates of interpersonal emotion regulation and epigenetic underpinnings is also not yet understood. In adults with BED, different treatment options, lengths and applications result in at least moderate to high effects for the reduction of binge eating, whereas the efficacy of interventions aiming at LOC or BED in youth is still under studied.<br>In order to clarify the role of interpersonal emotion regulation problems, we propose a combined cross and longitudinal questionnaire based and an experimental laboratory approach. We plan to examine the interrelations of social exclusion experiences and rejection sensitivity with emotion regulation problems, negative mood and negative affect, eating disorder pathology, body weight, gaze behavior and psychophysiological correlates. We also aim to increase availability of and access to treatment for youth and evaluate the specific and additive efficacy of a traditional cognitive behavioral treatment approach and of interventions targeting at interpersonal emotion regulation problems. For the first time, in the area of LOC and BED, we will explore the role of epigenetic features prior and during treatment.<br>*See study protocol p. 2-6.*<br>Benefits are the knowledge gain regarding the development and maintenance of LOC in youth and the availability of evidence-based treatment in near future. Potential harms include a potential lack of benefit when participating in the online treatment trial. The study team will supervise the treatment course of each participant carefully and intervene during and after treatment where necessary. The pilot treatment trial did not reveal negative effects.<br>*See study protocol p. 15.*

	6b	Explanation for choice of comparators

Each of the three different substudies aims at contributing to a better understanding of the factors which lead to and maintain LOC and to an improved access to an evidence-based treatment in youth.

See comparators in additional materials to the study protocol, Table 1.

Study 1: The overall goal of study 1 is to examine cross-sectional and longitudinal associations between adverse social experiences such as social exclusion, rejection sensitivity, emotion regulation problems, mood, LOC and eating disorder pathology in youth. Study 1 consists of two substudies: a questionnaire-based study and an App-based daily-life study.

Primary outcome of the questionnaire-based study:

- Association of rejection sensitivity with eating disorder pathology (eat, weight and shape concern, restraint eating, and emotional eating; drive for muscularity and excessive exercising; urge to eat), with the number or severity of weekly LOC episodes and with problems of emotion regulation at baseline.
- Association of rejection sensitivity with the number or severity of LOC episodes, with problems of emotion regulation and with eating disorder pathology at year 2
- Influence of increasing rejection sensitivity values on primary outcome effects
- To examine these associations, we will assess questionnaire-based data of LOC, eating disorder pathology and rejection sensitivity at baseline and one year later.

Primary outcome of the App-based daily-life study 1:

- Association of self-reported rejection experiences in daily life with negative affect, emotional eating and LOC episodes and dysfunctional emotion regulation across both groups LOC and the HCG
- Influence of increasing rejection sensitivity values on primary outcome effects

Study 2: The overall goal of study 2 is to examine psychological, physiological and epigenetic correlates of rejection sensitivity and effects of social exclusion in youth with LOC and healthy controls. The experimental induction of social exclusion and rejection sensitivity relies on the Cyberball paradigm (Hartgerink et al., 2015; Rosenbach & Renneberg, 2011; Williams & Jarvis, 2006), where the participant is excluded from a computerized ball-toss game by two avatars (Hartgerink et al., 2015).

Primary outcome of study 2 (VR Cyberball game):

- social threat-related effects during (lower HRV) and after the exclusion (lower HRV; increased negative/reduced positive affect; impairment of basic needs, impaired subjective and objective inhibition) and a delayed recovery in youth with LOC compared to the HCG (lower HRV)
- Influence of increasing rejection sensitivity values on primary outcome effects and urge to engage in disinhibited eating in comparison with the

Primary outcomes of the study 3 i-BEAT trial:

- Reduction of the number or the severity of weekly LOC episodes, problems of emotion regulation, the eating disorder pathology and negative mood from preto post treatment and in comparison with a 4-weeks' waiting period.
- Reduction of weekly rejection sensitivity experiences, instances of problematic emotion regulation and negative mood in the INTER-E module in comparison with the CBT-E module.
- Reduction of the number or the severity of weekly LOC episodes (wLOC) and urge to engage in disinhibited eating in comparison with the interpersonal emotion regulation module.
- Further improvement of rejection sensitivity experiences, emotion regulation capacities, number and severity of weekly LOC episodes and urge to engage in disinhibited eating of applying additional CBT-E or INTER-E module and leveling of initial treatment specific effects.

Objectives	7	Specific objectives or hypotheses

See study protocol p. 6-9.

Trial design	8	Description of trial design including type of trial (eg, parallel group, crossover, factorial, single group), allocation ratio, and framework (eg, superiority, equivalence, noninferiority, exploratory)<br>Based on the pilot study BEAT (primary register number: DRKS00014580), this research program will investigate etiological factors of Loss of Control Eating (LOC) in youth applying a mixed longitudinal and cross-sectional questionnaire survey and experimental study with two waves (T0 + second assessment after one year and an App-based daily life assessment (study 1) as well as a VR study to assess social exclusion and social rejection sensitivity (study 2). VR increases the ecological validity of the assessment of social rejection sensitivity (increases feeling of reality and to be part of the Cyberball paradigm). VR furthermore allows assessing self-reported feelings immediately during the task as well as the assessment of eye-gaze, posture and physiological correlates (heart rate variability, HRV) in male and female youth with LOC. A gamified stop signal task (SST) procedure allows an engaging and objective approach to inhibition and thus impulsivity measures in the context of binge eating BE and LOC eating in youth. In addition, based on our experiences during the treatment pilot study including three age-adapted face-to-face group-workshops and 6 standardized email-guided self-help sessions (DRKS00014580), we improved treatment content by delivering treatment with 6 state of the art CBT-E treatment sessions for Binge Eating Disorder and LOC and 6 Interpersonal emotion regulation sessions in order to assess additional effects and superiority of one of the two modules. Accessibility of the treatment content which was previously delivered during the workshops is further improved by using a modified and streamlined version of our online treatment platform (Munsch et al., 2019a) (i-BEAT, study 3).<br>Study 2 and 3 of the i-BEAT trial are designed as a randomized controlled single center study and the treatment trial includes two parallel groups with the primary endpoint of number of LOCs among others. Randomization will be performed as block randomization with a 1:1 allocation.<br>*See study protocol p.6. superiority effects of one treatment type.*

Methods: Participants, interventions, and outcomes

Study setting	9	Description of study settings (eg, community clinic, academic hospital) and list of countries where data will be collected. Reference to where list of study sites can be obtained<br>i-BEAT is a single-site academic institution (Department of Psychology, University of Fribourg, Switzerland / outpatient clinic-based (lead: Simone Munsch) trial, which will recruit and treat mostly online via social media and in German speaking schools, professional schools and universities.<br>*See study protocol p.11.*

Eligibility criteria	10	Inclusion and exclusion criteria for participants. If applicable, eligibility criteria for study centres and individuals who will perform the interventions (eg, surgeons, psychotherapists)<br>Participants' inclusion criteria for studies 1-3 are age between 14 and 24 years, sufficient German language competences and written informed consent. Criteria for LOC are fulfilled and youth included if they experience at least 3 episodes of LOC during the last 3 months accompanied by at least 3 out of 5 behavioral indicators and/ or some degree of distress, absence of absence of AN or BN. Inclusion criteria for youth participating in the HCG are healthy body weight (BMI 18.5-24.9), absence of any past or present ED and absence of any present mental disorder according to the diagnostic interview. Youth from the LOC group are excluded if they suffer from any current mental disorder preventing safe participation in the i-BEAT study or if there is an intake of weight affecting drugs, participation in an ED related psychotherapy or weight loss treatment. Females in pregnancy or lactation are excluded. Additional in- and exclusion criteria for study 2 are intact or corrected vision capacity and nausea in VR.<br>*See study protocol p.10.*<br>*For further information see Inclusion and additional inclusion criteria on https://www.drks.de/drks_web/setLocale_EN.do.*<br>All procedures will be performed by students of psychology or trained i-BEAT study staff members (psychologists, psychotherapists) under the supervision of FF, MR and SM.
Interventions	11a	Interventions for each group with sufficient detail to allow replication, including how and when they will be administered<br>Randomization will be performed as block randomization with a 1:1 allocation.<br>*See Table 1 for more information on the time-schedule of the study and study protocol p.11-13 for procedures and interventions of the Cyberball study as well as Table 2 in the study protocol for information on content and procedure during the treatment study.*
	11b	Criteria for discontinuing or modifying allocated interventions for a given trial participant (eg, drug dose change in response to harms, participant request, or improving/worsening disease)<br>Allocation to any aspect of the i-BEAT trial can be discontinued if the participant wishes for. A modification of the sequence of allocation to either CBT-E or INTER-E module first is not possible.

	11c	Strategies to improve adherence to intervention protocols, and any procedures for monitoring adherence (eg, drug tablet return, laboratory tests)<br>We established a Website (https://www.unifr.ch/psycho/de/i-beat/home/) and an Instagram account (https://www.instagram.com/ibeat_unifr/), where we continuously inform about the study development and provide information on mental well-being etc. We expect that this contact reinforces adherence. During waiting for treatment access, participants receive short e-mails, informing them about the remaining waiting time. Online treatment is assisted by an individual coach, who is contacted weekly and who provides individual feed-back on tasks performance and questions of the treatment trial participants.<br>*See study protocol p.12-13.*
	11d	Relevant concomitant care and interventions that are permitted or prohibited during the trial<br>Youth from the LOC group are excluded if they suffer from any current mental disorder preventing safe participation in the i-BEAT study or if there is an intake of weight affecting drugs, participation in an ED related psychotherapy or weight loss treatment.<br>*See study protocol p.10 and Table 3.*
Outcomes	12	Primary, secondary, and other outcomes, including the specific measurement variable (eg, systolic blood pressure), analysis metric (eg, change from baseline, final value, time to event), method of aggregation (eg, median, proportion), and time point for each outcome. Explanation of the clinical relevance of chosen efficacy and harm outcomes is strongly recommended<br>A detailed reporting on these questions can be found on the DRKS Webpage related to our project:<br>https://www.drks.de/drks_web/setLocale_EN.do
Participant timeline	13	Time schedule of enrolment, interventions (including any run-ins and washouts), assessments, and visits for participants. A schematic diagram is highly recommended (see Figure)<br>As the study's recruitment is only beginning, we did not yet establish such figures.

Sample size	14	Estimated number of participants needed to achieve study objectives and how it was determined, including clinical and statistical assumptions supporting any sample size calculations<br>For study 1 we will recruit N= 600 youth and expect a participation rate of c. 70-80% after one year. Power is sufficient (1-b=.8) to detect small to medium effect sizes f2 of c. 0.08, applying multiple regression models including covariates and testing specific predictors, for a given b of .05. For the App based study 1 multilevel models are applied to carry out between-subjects analysis of covariance. Moderators will be included to test for the interaction effects between groups (LOC vs HCG) within the Cyberball task. We expect moderate to large effect sizes (d=0.8) with sufficient power to detect significant effects given 1-b=.8 and a=.05 for study 2 with N=60 youths, even when accounting for c. 13% dropouts. For study 3 mixed between and within-subjects analysis of covariance or linear mixed models are carried out. Based on our BEAT pilot trial (DRKS00014580), where a high effect size for within subject measures revealed (d=1.37) a medium to high effect size for these effects can be assumed (f=0.25, taking r=0.5 for the correlation among repeated measures), the required sample size would be N=34 with sufficient power to detect significant effects given 1-b=.8 and b=.05 for study 3 with N=120 youths, even when accounting for dropouts. Exploratory epigenetic goals involve predictors and within-subject effects and are expected to be sufficiently powered if effect sizes are medium to large.<br>*See study protocol p.10-11.*
Recruitment	15	Strategies for achieving adequate participant enrolment to reach target sample size<br>Randomization will be performed as block randomization with a 1:1 allocation.<br>*See study protocol p.6. superiority effects of one treatment type.*

Methods: Assignment of interventions (for controlled trials)

Allocation:

Sequence generation	16a	Method of generating the allocation sequence (eg, computer-generated random numbers), and list of any factors for stratification. To reduce predictability of a random sequence, details of any planned restriction (eg, blocking) should be provided in a separate document that is unavailable to those who enrol participants or assign interventions<br>Randomization according to blocking will be computer-generated. There will not be any factor for stratification. Randomization is supervised and provided by FF (post doc student).<br>*See study protocol p.10.*

Allocation concealment mechanism	16b	Mechanism of implementing the allocation sequence (eg, central telephone; sequentially numbered, opaque, sealed envelopes), describing any steps to conceal the sequence until interventions are assigned<br>During informed consent procedure, the study participants are informed about the randomized allocation to the CBT-E or INTER-E first treatment and that the other module will follow thereafter. As soon as the participants access the first therapy session, they are informed about the type of therapy presented to them first.
Implementation	16c	Who will generate the allocation sequence, who will enrol participants, and who will assign participants to interventions<br>The allocation sequence is generated automatically on the treatment platform. Participants are enrolled via the webpage and assigned by VM to the study 1, AN to study 2 and by FF to the treatment trial.
Blinding (masking)	17a	Who will be blinded after assignment to interventions (eg, trial participants, care providers, outcome assessors, data analysts), and how<br>As soon as participants enter the treatment phase, there is no blinding.
	17b	If blinded, circumstances under which unblinding is permissible, and procedure for revealing a participant's allocated intervention during the trial<br>Not applicable.

Methods: Data collection, management, and analysis

Data collection methods	18a	Plans for assessment and collection of outcome, baseline, and other trial data, including any related processes to promote data quality (eg, duplicate measurements, training of assessors) and a description of study instruments (eg, questionnaires, laboratory tests) along with their reliability and validity, if known. Reference to where data collection forms can be found, if not in the protocol<br>Study procedure as well as instruments are described in detail in the *additional files, Table 1.*
	18b	Plans to promote participant retention and complete follow-up, including list of any outcome data to be collected for participants who discontinue or deviate from intervention protocols<br>Please refer to point 11b. Questionnaire-based data is assessed online via Qualtrics. The questionnaire asks for filling in all data before the next page can be accessed. Data assessment of participants who discontinue will include all available data up to this point. No further data assessment is feasible and therefore not planned.

Data management	19	Plans for data entry, coding, security, and storage, including any related processes to promote data quality (eg, double data entry; range checks for data values). Reference to where details of data management procedures can be found, if not in the protocol<br>Please refer to our data management plan, which has been mandatory to be provided during the funding process of the SNSF.<br>*See Appendix 1.*
Statistical methods	20a	Statistical methods for analysing primary and secondary outcomes. Reference to where other details of the statistical analysis plan can be found, if not in the protocol
	20b	Methods for any additional analyses (eg, subgroup and adjusted analyses)
	20c	Definition of analysis population relating to protocol non-adherence (eg, as randomised analysis), and any statistical methods to handle missing data (eg, multiple imputation)<br>20a-c:<br>Data will be analyzed with the Statistical Package of Social Sciences (SPSS) and R. We will apply multiple regression models including covariates and testing specific predictors (study 1), multilevel models including moderators (App based study 1), between subjects' analysis of covariance including moderators (study 2) and mixed between and within subjects analysis of covariance or linear mixed models (study 3). Exploratory epigenetic goals involve predictors and within-subject effects. Expected dropouts are assumed to amount up to 10–18%. Random occurrence should lead to equal distribution of dropouts in the different groups (LOC vs. HCG; CBT-E first vs. INTER-E first). However, in the case of an unequal distribution of the number of participants to the different groups, this issue will be addressed with multi-level models. Multi-level models are assumed to be robust against violations of equal group sizes.[54] Additionally, we will test whether a missing at random pattern is a reasonable assumption with respect to dropout.<br>*See study protocol p.14.*
**Methods: Monitoring**		
Data monitoring	21a	Composition of data monitoring committee (DMC); summary of its role and reporting structure; statement of whether it is independent from the sponsor and competing interests; and reference to where further details about its charter can be found, if not in the protocol. Alternatively, an explanation of why a DMC is not needed<br>DMC are to members of the department of psychology, which are active in related but not in the area of clinical psychology, Prof. Petra Klumb, head of the ethical committee of the department and Prof. Jürgen Sauer, work and organizational psychology. DMC procedures were part of the ethical approval by the independent ethical committee of the canton of Berne, where all clinical trials and related testings have to be submitted.

	21b	Description of any interim analyses and stopping guidelines, including who will have access to these interim results and make the final decision to terminate the trial<br>Not applicable. In cases of severe critical incidence, the ethic committee of the canton of Berne has to be informed and cases as well as consequences for the study procedure are evaluated.
Harms	22	Plans for collecting, assessing, reporting, and managing solicited and spontaneously reported adverse events and other unintended effects of trial interventions or trial conduct<br>The ethical committee of the canton of Berne provides standardised Case Report Forms (CRFs), where such events are documented and yearly sent to the ethical committee. Serious adverse events have to be reported within the following 15 hours to the ethical committee.
Auditing	23	Frequency and procedures for auditing trial conduct, if any, and whether the process will be independent from investigators and the sponsor<br>The DMC will monitor the study procedure now at the beginning of the recruitment, then yearly and once at the end of the study. This planning has been submitted and approved by the cantonal ethic committee, Berne.

Ethics and dissemination

Research ethics approval	24	Plans for seeking research ethics committee/institutional review board (REC/IRB) approval<br>The final study protocol and procedures have been approved in April 2021.
	25	Plans for communicating important protocol modifications (eg, changes to eligibility criteria, outcomes, analyses) to relevant parties (eg, investigators, REC/IRBs, trial participants, trial registries, journals, regulators)<br>Any modification to the study protocol has to be announced to and evaluated by committee of the canton of Berne prior to the potential changes.
Consent or assent	26a	Who will obtain informed consent or assent from potential trial participants or authorised surrogates, and how (see Item 32)<br>According to the Swiss law, all participants aged 14 years and more are able to sign informed consent forms without informing authorized surrogates. We nevertheless invite our participants under 18 years (the age of 18 years marks full age according to the Swiss law) to inform their parents or surrogates about their participation. In cases of potential self-harm or danger to others we are obliged to inform surrogates and inform the participants accordingly, Appendix 2.

	26b	Additional consent provisions for collection and use of participant data and biological specimens in ancillary studies, if applicable<br>Epigenetic sampling is mentioned within the informed consent form, see Appendix 2.
Confidentiality	27	How personal information about potential and enrolled participants will be collected, shared, and maintained in order to protect confidentiality before, during, and after the trial<br>Trial and participant data will be handled with uttermost discretion and are only accessible to authorized personnel who require the data to fulfil their duties within the scope of the study. On the CRFs and other study specific documents, participants are only identified by a unique participant code (see chapter 8.2). There exists one list only, where the participants' names and other information to identify them are assigned to the code. This list is kept under lock at the department of psychology at the University of Fribourg (administrative office of the PI). The PI and members of the research group (see staff list appendix I) have access to these data for statistical analysis. All involved persons are committed to the duty of confidentially.<br>Audio files, generated during the DIPS in order to calculate interrater reliability values are handled with utmost care. In order to protect these sensitive data, the following measures are taken. Firstly, the audio files are stored on a password protected and access restricted server provided by the UNIFR (https://www.unifr.ch/it/de/speicherung-grosser-datenmengen.html). The restricted access is exclusively given to three people: The PI Prof. Simone Munsch, study staff member M.Sc. Adrian Naas and the UNIFR informatics personnel member of the department of psychology, Thierry Progin. Before getting access to the sensitive data folder, these three staff members have to accept and sign the confidentiality form. Furthermore, the audio files are coded before the upload. M.Sc. Adrian Naas will code the audio files before their upload and will be the only team member to know the key for the coding process. Audio files will only be generated from participants who gave written informed consent for the recording. Study staff sign a declaration of discretion before conducting the DIPS.<br>Epigenetic data in this study is not identified by participant name but by a unique participant code. Biological material is appropriately stored in a restricted area only accessible to the authorized personnel. Coding is applied to all biological data transmitted to our collaborator Prof. Robert Kumsta from the Ruhr-University of Bochum, a specialist lab regarding epigenetics and DNA analysis. At no point will Prof Kumsta and his team be able to decipher personal information of the genetic material that is being sent to them.<br>Data will be assessed and coded, so that the participant cannot be identified by any other person than the sponsor and the study organization (SM, FF, AN, VM). The list with code and names is kept under lock in the administrative office of the sponsor.

Declaration of interests	28	Financial and other competing interests for principal investigators for the overall trial and each study site<br>Not applicable.
Access to data	29	Statement of who will have access to the final trial dataset, and disclosure of contractual agreements that limit such access for investigators<br>The Sponsor and responsible person as well as staff members have access to the final data set in order to analyse and publish the findings.
Ancillary and post-trial care	30	Provisions, if any, for ancillary and post-trial care, and for compensation to those who suffer harm from trial participation<br>The insurance of the University of Fribourg will cover such compensation. This has been part of the evaluation of the i-BEAT trial by the ethical committee of the canton of Berne.
Dissemination policy	31a	Plans for investigators and sponsor to communicate trial results to participants, healthcare professionals, the public, and other relevant groups (eg, via publication, reporting in results databases, or other data sharing arrangements), including any publication restrictions<br>No publication restrictions. The SNSF requests open access publications and strongly recommends open data access.
	31b	Authorship eligibility guidelines and any intended use of professional writers<br>The authors will adhere to the APA eligibility guidelines (https://www.apa.org/research/responsible/publication). English language proof-editing might be necessary for some publications.
	31c	Plans, if any, for granting public access to the full protocol, participant-level dataset, and statistical code<br>None.
**Appendices**		
Informed consent materials	32	Model consent form and other related documentation given to participants and authorised surrogates<br>See Appendix 2
Biological specimens	33	Plans for collection, laboratory evaluation, and storage of biological specimens for genetic or molecular analysis in the current trial and for future use in ancillary studies, if applicable<br>See Appendix 3

*It is strongly recommended that this checklist be read in conjunction with the SPIRIT 2013 Explanation & Elaboration for important clarification on the items. Amendments to the protocol should be tracked and dated. The SPIRIT checklist is copyrighted by the SPIRIT Group under the Creative Commons "Attribution-NonCommercial-NoDerivs 3.0 Unported" license.

Table 3 | i-BEAT trial registration data

Data category	Information [1]
Primary registry and trial identifying number	DRKS00023706
Date of registration in primary registry	27 November, 2020
Secondary identifying numbers	SNF identification number: 185387, ID of the cantonal ethics committee Berne: 2019-01277
Source(s) of monetary or material support	Swiss National Fonds (SNF; http://p3.snf.ch/Project-185387)
Primary sponsor	SNF
Secondary sponsor(s)	Not applicable
Contact for public queries	i-BEAT@unifr.ch
Contact for scientific queries	Simone Munsch, simone.munsch@unifr.ch; i-BEAT@unifr.ch
Public title	i-BEAT: Binge-Eating Adolescent and Young Adults Treatment – ein Forschungs- und Internet-basiertes Behandlungs-programm für Jugendliche und junge Erwachsene mit Essanfällen
Scientific title	i-BEAT: Binge-Eating Adolescent and Young Adults Treatment – ein Forschungs- und Internet-basiertes Behandlungs-programm für Jugendliche und junge Erwachsene mit Essanfällen
Country of recruitment	Switzerland
Health condition(s) or problem(s) studied	Binge Eating Disorder (BED) and loss of control eating (LOC)
Intervention Arm 1 (Study 3)	Experimental condition: Internet-based self-help treatment targeting interpersonal emotion regulation problems
	Control condition: Traditional cognitive behavioral treatment (CBT-E)
Intervention Arm 2 (Study 2)	Experimental condition: Youth with and without BED / LOC eating experiencing the social exclusion condition (ostracism) of the Cyberball task
	Control condition: Youth with and without BED / LOC eating experiencing the social exclusion inclusion (ostracism) of the Cyberball task
Intervention Arm 3 (Study 1)	Experimental condition vs. control condition: Not applicable as the screening study 1 constitutes a cross-sectional screening study with no active or placebo conditions.
	Experimental condition vs. control condition: Not applicable as the app-based daily life study 1 examines effects of different (e.g. emotional) states on reported eating disorder behaviours.
Key inclusion and exclusion criteria	Ages eligible for study: 14 – 24 years; Sexes eligible for study: both; Accepts healthy volunteers: yes
	**Inclusion criteria BED/LOC group:**<br>- Willingness and self-reported motivation to participate.<br>- Full diagnosis of BED according to the DSM-5.<br>- Subclinical BED or LOC (experiencing loss of control during food intake which is <u>not</u> accompanied by the consumption of objectively large amounts of food. Frequency: At least 3 binge eating episodes or LOC episodes during the last 3 months, accompanied by at least 3 of 5 Behavioural BED indicators (according to DSM-5) and/or distress.<br>- Informed consent by signature: Patients must agree to the study consent form (Study 1: screening and/or app-based daily life study 1, and/or Study 2: VR Cyberball paradigm and/or epigenetics, and/or Study 3: treatment study).
	**Inclusion criteria healthy control group (HCG):**<br>- Willingness and self-reported motivation to participate.<br>- Healthy body weight (BMI 18.5 – 24.9).<br>- No previous or current eating disorder and no current mental disorder.<br>- Informed consent by signature: Participants of the healthy control group must agree to the study consent form (Study 1: questionnaire-based and/or app-based daily life study 1, and/or Study 2: VR Cyberball paradigm and/or epigenetics).
	**Exclusion criteria:**<br>- Language proficiency: The i-BEAT study is performed in German and inadequate language proficiency leads to study exclusion.<br>- Current or past mental disorder.<br>- Pregnancy (app-based daily life study 1, study 2 and 3). It is possible to participate in the screening study 1 with pregnancy.<br>- Previous participation in i-BEAT.<br>**Additional exclusion criteria study 2**<br>- Participants must have intact or corrected vision.<br>- Participants are excluded if they report nausea in VR (motion sickness).
Study type	Multimethod / Interventional
	Allocation Study 1: Not applicable<br>Allocation Study 2<br>- Allocation Participants (BES/LOC vs. HCG): Pseudo-experimentally<br>- Cyberball conditions are controlled for longitudinally within subjects in fixed order (exclusion condition first)<br>Allocation Study 3: Randomized controlled trial
	Primary purpose Study 1: Screening of the general population between 14 – 24 years and participant identification for studies 2 and 3. Creating awareness for BED in the general population.<br>Primary purpose Study 2: Evaluating differences in reaction to social exclusion between BES/LOC participants and a HCG.<br>Primary purpose Study 3: Examination of the online INTER-E therapy effects in comparison to traditional online CBT-E therapy in BED/LOC participants.
Date of first enrolment	April 2021
Target sample size	Screening study 1: 600<br>App-based daily life study 1: 60<br>Study 2: 60<br>Study 3: 120
Recruitment status	Recruiting
Primary outcome(s)<br>Note, a complete and more detailed overview of outcomes examined during the study at hand is given in the following document:<br>Additional file table 1	**Study 1:** Investigates cross-sectional and longitudinal associations between social exclusion, rejection sensitivity, social phobia, emotion regulation, mood, beauty ideal, inhibition capacities and eating disorder pathology such as LOC in youth. Additionally, epigenetic correlates of rejection sensitivity are assessed.<br>**Study 2:** Examines psychological and physiological effects during and after social exclusion in youth with BED/LOC and healthy controls in the laboratory. During the process, affect, urge to eat, heart rate variability, impairment of basic needs, inhibition capacities, eye tracking and recovery from ostracism are assessed.<br>**Study 3:** Assesses the differential and additive efficacy of altogether 15 sessions CBT-E and interpersonal emotion regulation (INTER-E) online intervention to treat BED/LOC and interpersonal difficulties (such as rejection sensitivity and effects of social exclusion). The number and severity of weekly LOC episodes, general eating disorder pathology, mood, inhibition capacities, emotion regulation, weekly rejection experiences and urge to eat are assessed weekly or before and after the treatment modules (CBT-E and INTER-E).
Key secondary outcomes	Study 1: Though shape fusion, body mass index (BMI), body dissatisfaction, emotional eating.<br>Study 2: Rejection sensitivity, negative evaluation of ambiguous social scenarios, distress, pupillary dilation, hyperscanning and social gaze aversion (eye tracking) and hand posture.<br>Study 3: Stability of the proposed therapy effects, therapists' clinical general impressions.

1. i-BEAT: Binge-Eating Adolescent and Young Adults Treatment – ein Forschungs- und Internet-basiertes Behandlungs-programm für Jugendliche und junge Erwachsene mit Essanfällen [https://www.drks.de/drks_web/navigate.do?navigationId=trial.HTML&TRIAL_ID=DRKS00023706] Accessed on 16 August 2021

www.snf.ch
Wildhainweg 3, Postfach 8232, CH-3001 Bern

Data management plan (DMP)

1 Data collection and documentation

1.1 What data will you collect, observe, generate or reuse?

Data generated in the study
data type; form; content; volume

Multiple occasion assessment (EMA) over a period of two weeks:
investigates time-lagged associations between ER skills application (self-report), food-related cognitive control (n-back test) and subsequent binge episodes (self-report) in BED and matched obese and normal weight samples.

Laboratory tasks:
Data will include self-reports assessed via visual analogue scales (VAS) and arousal as well as indicates of valence and type of emotion coded via Facet.

Volumes: flat file in ASCII format, covering between 60 and several hundred rows and 50–100 columns each (cave: the DFG, where the main applicant submitted the proposal initially does not ask for DMP details prior to evaluation. Thus, specifications and adaption might be necessary to satisfy both DFG and SNSF requirements).

Up to now, it is not planned to include external or own data from prior research

1.2 How will the data be collected, observed or generated?

We will run a daily life and a laboratory study to generate data.
Quality standards and assurance:
Quality assurance regarding the experimental designs is implemented by different types of interventions. First, all study collaborator (PhD students and Psychology students) are trained initially and repeatedly in terms of a standardized conduction of the experimental procedure both in the EMA and especially in the laboratory study. We further train and guide the collaborators when in comes to dealing with difficulties and emergencies. To this end, several research meetings of the German and the Swiss group have been planned and put on the budget accordingly. Further quality assurance and quality control systems will be implemented and maintained with written SOPs and working instructions. All technical, experimental and psychotherapeutical procedures will be carried out according to standardized protocols, which will be available for all trainers at any time.

Data storage:
Data is stored in ASCII-format on a virtual server provided by the University of Fribourg. In the present study the web Framework Laravel with the database system MySQL will be used. This software stack is similar to the one used by REDCap and fulfils the standards of data security and traceability of database's changes (HFG-conform according to KlinV Art.18). Daily backups will be made. By default, the creation date and the last modification date of every entry in the database will be stored. Moreover, the history of changes and the user ID of the person who made the change will be stored.

1.3 What documentation and metadata will you provide with the data?

Meta data including full description of variables and versions will be generated in separate SPSS or R scripts and electronically stored in a separate file.

An additional file includes sources of data: recorded date of questionnaire and interview assessments and diagnosis, date and participation in experimental paradigm, fulfillment of inclusion criteria, data according to the CRF, demographic data, informed consent sheets, occurrence of AEs (patient's mental health decreases because of other than BED condition) and concomitant procedure. Source data is kept under lock in the administrative office of the applicants (Jen Svaldi (lead, G)/ Simone Munsch (CH)).

2 Ethics, legal and security issues

2.1 How will ethical issues be addressed and handled?

Data Safety standards and procedures:
Participants receive a code, which is used in relation to all data. Paper-pencil questionnaires that will be distributed, protocols of the diagnostic structured interviews as well as other documents (e.g. informed consents) will be kept under lock (coded) in folders and/or in the trial master file (TMF) in the administrative office of the PIs (Jen Svaldi, G, lead; Simone Munsch, Swiss project partner). Data from paper-pencil questionnaires and protocols of diagnostic interviews will be transferred into an electronic data file (SPSS) that will be stored on the server of the University of Fribourg (Swiss project partner). The data is archived for 10 years.

All participants are informed that they can drop out at any time and that the study group is at their disposition regarding advice or psychological support.

Permission to carry out the research: The cantonal ethical committee (Bern) as well as the ethical committee of the University of Tübingen approved the originally submitted study.

2.2 How will data access and security be managed?

All data (self-report and behaviorall) collected during the study will be coded and stored password protected on the server of the University of Fribourg (Swiss project partner). This means that name, address, date of birth are replaced by a number which is in no way related to the before mentioned personal data. There exists one list, where the participants' names and other information to identify them are assigned to the code. This list is kept under lock at the department of psychology at the University of Fribourg (administrative office of the PI). All study data are archived for a minimum of 10 years after study termination or premature termination.

The PI and members of the research group have access to these data for statistical analysis. All involved persons are committed to the duty of confidentially. Direct access to source documents will be permitted for purposes of monitoring, audits and inspections. The PI, the members of the Internal review board for research ethics and monitoring (IRB) and the data safety monitoring committee (DSMC) of the department, the statistician (Dr. Andrea Hans Meyer, University of Basel) and the study management (PI) will have access to protocol, dataset, statistical code, etc. during and after the study. The PI, the statistician, the study management will use the data for dissemination of the study findings.

2.3 How will you handle copyright and Intellectual Property Rights issues?

The owner of the data are the the University of Fribourg, Department of Psychology with the applicant Simone Munsch (Swiss project partner) and the University of Tübingen (German project partner: lead).

At the point of the beginning of the project, neither licenses nor the inclusion of external data are planned. This point will be updated at the end of the project grant as requested.

3 Data storage and preservation

3.1 How will your data be stored and backed-up during the research?

Data are stored in several data files in ASCII-format which can be merged at any time to analyse the variables required. Data files only contain raw data, i.e. no composite variables such as index variables or scales based on individual items. Composite variables will be centrally computed using SPSS-syntax files or R-script files. All variables being numeric will be validated, i.e. those of types continuous (including dates), ordinal, and nominal. Validation basically comprises the detection of improbable (e.g. outliers) or impossible values. To this end, single-variable and cross-variable checks will be performed. Validation rules will be stored in SPSS-syntax or R-script files. Our statistician (Dr. Andrea Meyer) will be appointed persons responsible for data validation and correct data dissemination. Data preparation before analysis involves the identification of univariate (z-scores) and multivariate outliers (Cook's distances). Continuously distributed outcome variables will be checked for normality and homoscedasticity and transformed if necessary prior to any analysis.

3.2 What is your data preservation plan?

Data are stored in an ASCII format on the local server of the University of Fribourg and daily backups are made.

4 Data sharing and reuse

4.1 How and where will the data be shared?

Open access data policies are respected and data will be shared via suggests data repositories (FAIR Data Principles). Please consider, that the concrete procedure has to be adapted to the DFG requirements (lead: Prof. Jennifer Svaldi).

4.2 Are there any necessary limitations to protect sensitive data?

All completely anonymised data will be provided at the time point of the publication of a specific research question. (Please respect that details of the procedure might have to be adapted according to the requirements of the DFG).

4.3 All digital repositories I will choose are conform to the FAIR Data Principles.

Yes

4.4 I will choose digital repositories maintained by a non-profit organisation.

Yes

D1

Short title: i-BEAT - Binge-Eating Adolescent And Young Adults Treatment - a research and internet-based treatment programme for adolescents and young adults with binge eating disorders

Project management: Prof. Dr. Simone Munsch, University of Fribourg, Switzerland

Dear participant,

Welcome to our survey on the experience of social situations and eating behaviour. Thank you for your interest in this study. The aim of the study is to investigate the relationship between perceived rejection and the occurrence of binge eating. The study is open to adolescents and young adults aged 14-24 years, whether or not they experience binge eating. Adolescents/young adults who are pregnant cannot participate. We will not conduct a pregnancy test, but will rely on the information provided by the participants. Also excluded from participation are adolescents and young adults who are in an employment relationship with Prof. Munsch or the working group of the Chair of Prof. Munsch or who work for Prof. Munsch.
We would like to ask you to answer a few questions on these topics below and to complete a short socio-demographic questionnaire. Following this questionnaire study, you will have the opportunity to participate in further studies in the laboratory at the University of Fribourg, Switzerland. If you show signs of binge eating, you may also have the opportunity to participate in a treatment study for binge eating disorder.

1. Right

Participation in the study is voluntary and can be terminated at any time without justification. The study staff are subject to a duty of confidentiality towards third parties. This means that no information about your details will be passed on without your consent.

2. Data confidentiality

Personal and psychological data will be collected for this survey. Very few professionals will see the unencoded data, and this is solely to fulfil tasks within the study. When collecting data for study purposes, the data will be encrypted (coded). Encryption means that all personal data that could identify you (name, date of birth) will be deleted and replaced by a key/code. The key list always remains in the institution (University of Fribourg). People who do not know the key cannot therefore draw any conclusions about you. If the data are published, they will not give any clues about you and your name will never appear on the internet or in a publication. Sometimes a journal has a requirement that individual data (so-called raw data) must be submitted for publication. If individual data must be submitted, then the data is always encrypted and cannot be traced back to the person. All persons who have access to the data within the framework of the study are subject to the duty of confidentiality. Data protection regulations are adhered to and you, as a participant, have the right to view the data at any time. In order to make data readily available for future research purposes, we also have the option of making the encrypted individual data publicly accessible on a digital database. This has the advantage that other researchers have the opportunity to investigate new questions based on the data we have collected. However, we will only store your encrypted data, which do not allow any personal details (name, place of residence), on a digital database if you agree to this (see below).

3. Compensation for participants

Participation in this survey is not remunerated. Participation in the study does not entitle you to any commercial developments (e.g. patents). Psychology students at the University of Fribourg/Freiburg are remunerated for their participation by means of subject hours.

4. Funding of the study

The study is funded by the Swiss National Science Foundation (SNF 10001C_185387 / 1).

5. Contact person(s)

If you have any questions or uncertainties that arise during or after the survey, you can always contact one of these contact persons:

Prof. Dr. Simone Munsch
Chair of Clinical Psychology and Psychotherapy
Department of Psychology
University of Fribourg

M.sc. Felicitas Forrer
Graduate assistant
RM 01 bu. C-2.118
Rue P.A. de Faucigny 2

UNIVERSITÉ DE FRIBOURG
UNIVERSITÄT FREIBURG

Rue de Faucigny 2
CH-1700 Fribourg
Tel. Prof. Dr. Simone Munsch: + 41 26 300 76 55
Email: simone.munsch@unifr.ch

CH-1700 Fribourg
Tel. +41 26 300 7658
Email: felicitas.forrer@unifr.ch

M.Sc. Verena Müller
SNF doctoral student
RM 01 bu. C-2.118
Rue P.A. de Faucigny 2
CH-1700 Fribourg
Phone M.Sc. Verena Müller: +41 26 300 7659
Email: verena.mueller@unifr.ch

M.Sc. Adrian Naas
SNSF doctoral student
RM 01 bu. C-2.113
Rue P.A. de Faucigny 2
CH-1700 Fribourg
Phone M.Sc. Adrian Naas: +41 26 300 7478
Email: adrian.naas@unifr.ch

Please click:

☐ I have been informed in writing about this survey and agree to participate. I have read and understood all the information in full. Enclosed is my email address for further contact:

☐ I agree with the described collection and processing of the socio-demographic data and the questionnaires.

☐ Following the questionnaire study, I would like to be contacted and informed about the possibility of participating in another study in the virtual laboratory and about a study on the treatment of binge eating via the internet.

Please click:

I consent to my fully encrypted data being made publicly available on a digital database for research purposes.

☐ Yes No☐

I study psychology at the University of Fribourg/Freiburg and am therefore remunerated by means of subject hours.

☐ Yes No☐

D2

Short title: i-BEAT - Binge-Eating Adolescent and Young Adults Treatment - a research and internet-based treatment programme for adolescents and young adults with binge eating disorders

Study information for adolescent participants with binge eating disorder

Project management: Prof. Dr. Simone Munsch, University of Fribourg, Switzerland

Dear young person

We would like to ask you if you would like to participate in a clinical trial. In the following, these study projects are presented to you.

More detailed information

1. Aim of the studies

There are four studies in this project: "i-BEAT App", "i-BEAT VR", "i-BEAT Online" and "i-BEAT Epigenetics". With these we want to investigate different things:
- "i-BEAT App": Here we would like to learn more about the experience of social exclusion and its effects on mood and eating behaviour in adolescents and young adults with binge-eating disorder (BES: binge eating disorder) and/or regular loss of control, with the help of a smartphone app.
- "i-BEAT VR": In this laboratory study, we investigate the psychological and physiological effects of an interpersonal situation in virtual reality (VR).
- "i-BEAT Online": Here we are investigating the effectiveness of an internet-based treatment programme for the therapy of BES and/or regular loss of control over eating in adolescents and young adults.
- "i-BEAT Epigenetics": Here we take a saliva sample through which we can examine how experiences in everyday life can affect the expression of genetic traits.

What all the studies have in common is that we want to use them to better understand how loss of control over eating occurs, what perpetuates this problem and how it can best be treated. While "i-BEAT App" focuses on the personal experience in everyday life, with "i-BEAT VR" and "i-BEAT Epigenetics" we are investigating even more closely how the experience of loss of control when eating is related to physical measures (e.g. the heartbeat) and the expression of genetic characteristics. With "i-BEAT online" we are trying to better understand how to optimally treat the issue.

2. Selection

This study is open to all adolescents/young adults between the ages of 14 and 24 who suffer from binge eating disorder (BES), regular binge eating and/or a feeling of loss of control over food, as well as people without these characteristics as a comparison group. You will also need a smartphone, internet access to receive emails and a good enough knowledge of German to participate in one of the studies. Adolescents/young adults who also suffer from another serious mental illness that needs to be treated first or adolescents/young adults who are pregnant cannot participate. We will not conduct a pregnancy test, but will rely on the information provided by the participants. Also excluded from participation are adolescents and young adults who are in an employment relationship with Prof. Munsch or the working group of the Chair of Prof. Munsch or who work for Prof. Munsch.

3. General information

This study is being conducted by the University of Fribourg, Switzerland, under the direction of Prof. Dr. Simone Munsch. With the help of the internet questionnaire that you filled out, we were already able to gather initial information about your situation. Next, a more detailed interview will take place by telephone, which will last about an hour. Afterwards, you can take part in one or more of the studies described. Participation in the different studies takes different amounts of time and takes place at different times.

"**i-BEAT App**" will last a total of 7 days. A total of 60 people will take part in this study. You will be asked to report on your experiences and how you are feeling 3 times a day with the help of a smartphone app. This will take about 30 minutes each day. This study can start in the next few days. If you take part in the "i-BEAT VR" study, we can use this opportunity to show you how to use the smartphone app. If you do not take part in i-BEAT VR, we will instruct you about this by phone or email.

"**i-BEAT VR**" will take place in the next few days at the University of Fribourg and will last about 2.5 hours. A total of 60 people will also take part in this study. You will participate in a social situation or a game in virtual reality (VR). We will also measure your heartbeat and eye movements, which is not associated with any known side effects. In the course of this examination, we will also take a photo of you, which will be deleted at the end of the examination.

"**i-BEAT Online**" will start in about 4 weeks and last about 15 weeks from then on. A total of 120 people will take part in this study. During the first 6 weeks of the study, you will participate in an internet-based therapy. After a 3-week break, you will take part in another 6-week therapy. One of the two therapies focuses on difficulties with eating, while the other therapy focuses on relationships with other people. It is randomly decided which of the two therapies to start with (i.e. both options are chosen with a probability of 50%). The treatment is based on a tested therapy programme for adults, which has now been adapted for adolescents/young adults. Here, on the one hand, content is conveyed via the internet, while on the other hand, there is also a weekly exchange with a trained therapist via the news portal of an online platform.
Alternatively, it is possible to take up treatment at the psychotherapeutic practice centre of the University of Fribourg at regular conditions.

"**i-BEAT Epigenetics**" can take place as part of the "i-BEAT VR" study, if you take part in it. Then we will ask you to take a swab from your cheek mucosa with a cotton swab. If you do not take part in "i-BEAT VR", we will send you cotton swabs by post and ask you to send them back to us after the swab has been taken. A total of 60 people will also take part in this study. The investigation of these epigenetic processes does not allow any conclusions to be drawn about hereditary diseases, but we are investigating how environmental influences affect the hereditary dispositions, i.e. the reading of the genes.

In addition, we will ask you in about a year's time to fill out the questionnaire on the internet again. This way we can better understand how your situation has changed during this time.

We conduct this study in accordance with the laws in Switzerland. We also comply with all internationally recognised guidelines. The responsible cantonal ethics committee has reviewed and approved the study. You can also find a description of this study on the website of the Federal Office of Public Health: www.kofam.ch.

4. Procedure

After you have given your written consent to participate in the study, you will be given an appointment for a one-hour diagnostic interview over the phone. This interview will allow us to check whether you meet the criteria for participation in the study and whether the treatment programme is suitable for you. If so, we will invite you to participate voluntarily in one or more of the studies mentioned at the University of Fribourg, Department of Psychology[1].

Your contact person in Fribourg, who will accompany you through the experimental examinations and take the buccal swabs, is a student of clinical psychology and psychotherapy who is trained by the study director. The treatment persons are trained psychologists at the Centre for Psychotherapy of the University of Fribourg and psychotherapists in advanced training who regularly consult with a psychotherapist certified by the Federal Office of Public Health (FOPH). The treatment persons are supported by Master's students from the Department of Clinical Psychology and Psychotherapy at the University of Fribourg. It will always be the same person who accompanies you through the entire treatment.

[1] Department of Psychology, University of Fribourg, Rue Faucigny 2, 1700 Fribourg.

You are free to discontinue your participation in the study at any time without any disadvantages for you. In rare cases, we may decide to exclude you from the study. This can happen if, in the course of the programme, we come to the conclusion that it would be better for you to take another or additional treatment in order to effectively reduce your burden. In this case, we would recommend a different or additional treatment programme. If you are in another treatment programme in addition to ours, this is not necessarily an exclusion criterion. However, adolescents and young adults who are concurrently enrolled in weight loss or eating disorder treatment cannot be included in the study.

5. Benefit

The treatment programme in the i-BEAT Online study is based on a proven treatment approach adapted to adolescents/young adults. The other questionnaire studies and laboratory studies are designed to understand how the experience of social exclusion, mood and loss of control over eating are related. In this regard, there is no immediate benefit or harm to be expected from your participation.

6. Right

You are participating in this study voluntarily. If you do not want to take part or later withdraw your participation, you do not have to justify this. With the exception of the questionnaire that you have just completed, participation in the experimental studies is not a prerequisite for participation in the treatment programme. Your psychological support is guaranteed regardless of your decision. You may ask questions about your participation in the study at any time. Please contact the persons named at the end of this information. Since the interview and the questionnaires are used to check the criteria for participation in the study, we may have to exclude you from the study if you do not meet the participation criteria. If the interview indicates that you have other mental health problems besides binge eating disorder that need treatment, you will be informed of this, subject to your consent, and you will receive information from us about further treatment options. The study staff are bound by the duty of confidentiality towards third parties, including your parents/guardians. This means that no information about the results of the interview and the course of the therapy will be passed on without your consent. Only in cases of acute danger to yourself (e.g. acute suicidal tendencies) or others will this confidentiality obligation be lifted and you and your parents/guardians will be informed by us and included in the emergency procedure (see Risks and burdens for participants).

7. Duties

As a participant, it is necessary that you adhere to the guidelines and requirements of this study (including completing the sessions according to the schedule, regular contact with the treatment person). The implementation of the therapy programme requires that you are in a sober state (not under the influence of psychoactive substances such as drugs or alcohol) and that you can devote yourself to the content of the sessions in a quiet place during the email-based sessions. You agree to report and inform us of any new symptoms, new complaints and changes in your condition if you are receiving other treatments and therapies at the same time.

8. Risks and burdens for the participants

For you as a participant in this study, there is no risk and no undesirable side effects are to be expected. In the course of this treatment, you will intensively deal with yourself and various problem areas, which can be a temporary burden. In addition, you will be continuously accompanied by a treatment person. In acutely stressful situations, an emergency procedure will be discussed and initiated. Information on crisis situations and behaviour as well as emergency contact details will be handed out to you at the time of the psycho-diagnostic interview.

When entering the virtual world with VR goggles in one of the experimental examinations, you may temporarily experience so-called motion sickness, the typical symptoms of which are dizziness or general malaise. If you experience any symptoms of motion sickness, we will immediately stop the examination in VR. You can also remove the VR glasses yourself at any time.

For adolescents/young adults of childbearing age: There are no risks and restrictions for adolescents/young adults of childbearing age. However, as pregnancy is associated with changes in eating habits, nutrition and body weight, these changes may affect the effectiveness of the programme. For this reason, if you are currently pregnant, we ask you to postpone your participation until 6 months after the birth.

9. Other treatment options

Apart from the treatment offered and to be reviewed in the study, no other therapy option is offered in the study. However, it is possible to participate in individual psychotherapy for the treatment of binge-eating disorder (binge eating disorder) at the Psychotherapeutic Practice Centre of the University of Fribourg (https://www3.unifr.ch/psycho/de/psychotherapie) instead of participating in the present treatment study (subject to a fee).

10. Results from the study

The study management will inform you during the study about any new findings that may affect the benefit of the study or your safety and therefore your consent to participate in the study. You will receive this information verbally and in writing.

11. Confidentiality of data and samples

Your personal, psychological and medical data will be collected for this study. Very few professionals will see your unencrypted data, and only to perform tasks within the study. When collecting data for study purposes, the data will be encrypted (coded). Encryption means that all personal data that could identify you (name, date of birth) are deleted and replaced by a key/code. The key list always remains in the institution (University of Fribourg). People who do not know the key cannot therefore draw any conclusions about you. If the results of the study are published, they will not reveal any information about you. Your name will never appear on the internet or in a publication. The individual data (so-called raw data) are made publicly accessible on a digital database so that they can be used for future research purposes. However, this data is always encrypted and can never be traced back to you as an individual. All persons who have access to your data in the course of the study are bound to professional secrecy. The data protection regulations are adhered to and you, as a participant, have the right to view your data at any time.
The buccal swabs are sent in encrypted form to a database/biobank in Bochum, Germany, where they are examined for this project and then immediately destroyed. The key list remains in the institution (University of Fribourg) and only the study management and the treatment staff have access to it. The study management is responsible for ensuring that the same standards are observed abroad as in Switzerland. The competent ethics committee may be given access to the data in order to check data security. The data may also be checked by the institution that initiated the study. The study director may have to disclose your personal data for such checks. All persons must maintain absolute confidentiality.

12. Resignation

You can stop and withdraw from the study at any time if you wish. The data and samples collected up to that point will continue to be evaluated in encrypted form, as information from people who do not want to or cannot complete the treatment is just as important for improving the treatment offered. After the evaluation, your data will be completely encrypted, i.e. your key allocation will be destroyed, so that no one can find out afterwards that the data originally came from you.

13. Compensation for participants

If you are not studying psychology at the University of Fribourg/Freiburg, your effort will be compensated in the following way. CHF 100 will be paid for participation in the "i-BEAT App" study and CHF 150 will be paid for participation in the "i-BEAT VR" study, including participation in the "i-BEAT Epigenetics" study. Your participation in the questionnaire, which you have just completed, will not be remunerated. If you study psychology at the University of Fribourg/Freiburg, you will receive subject hours for your participation in the studies "i-BEAT App", "i-BEAT VR" and "i-BEAT Epigenetics". Please take into account that students of psychology can compensate required subject hours by substitute performances (see Bachelor plan, page 17, point 9 : Experimental self-awareness www3.unifr.ch/psycho/en/assets/public/bachelor/plans/PLE_Psycho_BA180_De_rat 29012019v03.pdf). Participation in the treatment programme "i-BEAT Online" is free of charge. Expenses such as travel costs that are only incurred as a result of participation will be reimbursed to you in addition. Participation in the study does not entitle you to any commercial developments (e.g. patents).

14. Liability

The University of Fribourg and thus the project management by Prof. Simone Munsch, who initiated the study and is responsible for its implementation, is liable for any damage that might occur to you in connection with the research. The conditions and the procedure for this are regulated by law. For this purpose, the University of Fribourg has taken out insurance with the Basler Versicherungen in order to be able to cover liability in the event of any damage occurring during the performance of the experimental studies or the treatment programme. If you suffer any damage, you are requested to contact the head of the study, Prof. Simone Munsch.

15. Funding of the study

The study is funded by the Swiss National Science Foundation (SNF 10001C_185387 / 1).

16. Contact person(s)

If you have any questions, uncertainties or emergencies that arise during or after the study, you can contact one of these contact persons at any time:

Prof. Dr. Simone Munsch
Chair of Clinical Psychology and Psychotherapy
Department of Psychology
University of Fribourg
Rue de Faucigny 2
CH-1700 Fribourg
Tel. Prof. Dr. Simone Munsch: + 41 26 300 76 55
Email: simone.munsch@unifr.ch

M.Sc. Felicitas Forrer
Graduate assistant
RM 01 bu. C-2.118
Rue P.A. de Faucigny 2
CH-1700 Fribourg
Tel. +41 26 300 7658
Email: felicitas.forrer@unifr.ch

M.Sc. Verena Müller
SNF doctoral student
RM 01 bu. C-2.118
Rue P.A. de Faucigny 2
CH-1700 Fribourg
Tel. M.Sc. Verena Müller: +41 26 300 7659
Email: verena.mueller@unifr.ch

M.Sc. Adrian Naas
SNSF doctoral student
RM 01 bu. C-2.113
Rue P.A. de Faucigny 2
CH-1700 Fribourg
Tel. M.Sc. Adrian Naas: +41 26 300 7478
Email: adrian.naas@unifr.ch

In psychological emergency situations, you can also dial the 24-hour emergency number of the Swiss worry line (Dargebotene Hand): 143.

17. Glossary (terms requiring explanation):

Binge eating disorder: According to diagnostic criteria described in the Diagnostic and Statistical Manual of Mental Disorders (DSM-5, APA, 2013), this form of eating disorder is characterised by eating a large amount of food in a definable period of time and losing control over what and how much they eat. These so-called "eating attacks" are often accompanied by eating very quickly, in a jumbled manner, without hunger and to the point of an unpleasant feeling of fullness. As a result of these eating binges, feelings of shame, guilt and dejection set in.

Epigenetics: Field of biology and medicine that deals with which gene becomes active under which environmental influences. Epigenetic findings can, for example, help to understand under which biological and environmental conditions adolescents/young adults are more prone to binge eating.

If you would like to get more information or if other terms seem unclear, please visit our project homepage www.unifr.ch/go/i-BEAT. There we explain, for example, what emotion regulation is, what binge eating disorder (BES) is and how it differs from binge eating and loss of control eating, and what you can do against binge eating.

Declaration of consent

Written declaration of consent to participate in a study project

Please read this form carefully. Please ask if there is anything you do not understand or would like to know. Your written consent is required for participation.

**BASEC number (after submission):**	ID 2019-01277
**Study title:**	**i-BEAT**<br>Binge-Eating Adolescent and Young Adults Treatment<br>a research and internet-based treatment programme for adolescents and young adults with binge eating disorder.
**responsible institution:**	Prof. Dr. Simone Munsch<br>Chair of Clinical Psychology and Psychotherapy<br>Department of Psychology<br>University of Fribourg, Switzerland
**Place of implementation**:	University of Fribourg, Switzerland
**Responsible director of studies at the place of study:**	Prof. Dr. Simone Munsch<br>University of Fribourg, Chair of Clinical Psychology and Psychotherapy<br>Department of Psychology<br>University of Fribourg, Switzerland
**Participant:**<br>Name and first name in block capitals:<br><br>Date of birth:<br>Email address:<br>Gender:	 <br><br><br><br>☐ female ☐ male

- I have been informed verbally and in writing by the undersigned study director about the purpose, the procedure of the study, about possible advantages and disadvantages as well as about possible risks.

- I am voluntarily participating in this study and accept the content of the written information provided. I have had sufficient time to make my decision.
- My questions in connection with participation in this study have been answered. I will keep the written information and receive a copy of my written informed consent.
- I have been informed that the psycho-diagnostic interview and questionnaires at the beginning of the study will check the criteria for participation and that I may be excluded from the study if I do not meet the criteria.
- I was informed about possible other treatments and treatment procedures.
- I agree that the responsible experts of the study management and the responsible ethics committee may inspect my unencrypted data for testing and control purposes, but in strict compliance with confidentiality.
- I am informed of study results that directly affect my health.
  <u>If I do not wish to be informed, I inform the study director.</u>
- I know that my health-related and personal data (and samples) from this study can only be passed on in encrypted form for research purposes, including abroad.
- I can withdraw from the study participation at any time and without giving reasons. My further psychotherapeutic treatment is always guaranteed regardless of the study participation and of the participation in the experimental investigations. The data and samples collected up to the time of withdrawal will be used for the evaluation for the study.
- I am informed that the liability insurance of the University of Fribourg will cover any damages.
- I am aware that I must comply with the obligations stated in the participant information. In the interest of my health, the study management may exclude me from the study at any time.
- I am informed that participation in the experimental examinations in virtual reality and in the laboratory as well as the examination of biological factors are not a prerequisite for participation in the BEAT treatment programme.

<u>Please tick:</u>

I am taking part in the following/the following examinations:
☐ i-BEAT App
☐ i-BEAT VR
☐ i-BEAT Online
☐ i-BEAT Epigenetics

☐ I would like to be informed about the results of the psychodiagnostic examination and have been informed that in the event of acute mental states that are dangerous to myself or others, my parents/guardians will be informed and included in the emergency procedure.

☐ I do NOT wish to be informed about the results of the psychodiagnostic examination and have been informed that my wish will be respected, except in the case of acute mental conditions that are dangerous to myself or others.

☐ My parents/guardians should be informed about my participation and about the results of the psycho-diagnostic examinations in any case.

- Information to my parents after consultation with me will be in writing: ☐
- Information to my parents after consultation with me is verbal: ☐

Contact details of parents/guardians (informing parents about study participation if desired and in case of acute danger to self and/or others).

Name / First name	
Address	
Email address	

Telephone number	

Place, date	Signature Participant

Confirmation by the investigator: I hereby confirm that I have explained the nature, significance and scope of the study to this participant. I affirm that I will fulfil all obligations in connection with this study in accordance with applicable law. If, at any time during the conduct of the study, I learn of any issues that may affect the participant's willingness to participate in the study, I will inform him/her immediately.

☐ The adolescent's capacity to understand the content of the study and the effort involved in participating has been assessed (summary in the adolescent's own words) and is present.

Place, date	Surname and first name of the inspector in block capitals<br><br>Signature of the test person

D3

Short title: i-BEAT - Binge-Eating Adolescent and Young Adults Treatment - a research and internet-based treatment programme for adolescents and young adults with binge eating **disorder.**

Study information for young adults Participants with eating disorders

Project management: Prof. Dr. Simone Munsch, University of Fribourg, Switzerland

Dear participant,

We would like to ask you if you would like to participate in a clinical trial. In the following, these study projects are presented to you.

More detailed information

1. Aim of the studies

There are four studies in this project: "i-BEAT App", "i-BEAT VR", "i-BEAT Online" and "i-BEAT Epigenetics". With these we want to investigate different things:
- "i-BEAT App": Here we would like to learn more about the experience of social exclusion and its effects on mood and eating behaviour in adolescents and young adults with binge-eating disorder (BES: binge eating disorder) and/or regular loss of control, with the help of a smartphone app.
- "i-BEAT VR": In this laboratory study, we investigate the psychological and physiological effects of an interpersonal situation in virtual reality (VR).
- "i-BEAT Online": Here we are investigating the effectiveness of an internet and email-based treatment programme for the treatment of BES and/or regular loss of control eating in adolescents and young adults.
- "i-BEAT Epigenetics": Here we take a saliva sample through which we can examine how experiences in everyday life can affect the expression of genetic traits.

What all the studies have in common is that we want to use them to better understand how loss of control over eating occurs, what perpetuates this problem and how it can best be treated. While "i-BEAT App" focuses on the personal experience in everyday life, with "i-BEAT VR" and "i-BEAT Epigenetics" we are investigating even more closely how the experience of loss of control when eating is related to physical measures (e.g. the heartbeat) and the expression of genetic characteristics. With "i-BEAT Online" we are trying to better understand how to optimally treat the issue.

2. Selection

This study is open to all adolescents/young adults between the ages of 14 and 24 who suffer from binge eating disorder (BES), regular binge eating and/or a feeling of loss of control over food, as well as people without these characteristics as a comparison group. You will also need a smartphone, internet access to receive emails and a good enough knowledge of German to participate in one of the studies. Adolescents/young adults who also suffer from another serious mental illness that needs to be treated first or adolescents/young adults who are pregnant cannot participate. We will not conduct a pregnancy test, but will rely on the information provided by the participants. Also excluded from participation are adolescents and young adults who are in an employment relationship with Prof. Munsch or the working group of the Chair of Prof. Munsch or who work for Prof. Munsch.

3. General information

This study is being conducted by the University of Fribourg, Switzerland, under the direction of Prof. Dr. Simone Munsch. With the help of the internet questionnaire that you filled out, we were already able to gather initial information about your situation. Next, a more detailed interview will take place by telephone, which will last about an hour. Afterwards, you can take part in one or more of the studies described. Participation in studies takes different lengths of time and takes place at different times.

"i-BEAT App" will last a total of 7 days. A total of 60 people will take part in this study. You will be asked to report on your experiences and how you are feeling 3 times a day with the help of a smartphone app. This will

take about 30 minutes each day. This study can start in the next few days. If you take part in the "i-BEAT VR" study, we can use this opportunity to show you how to use the smartphone app. If you do not participate in i-BEAT VR, we will instruct you about this by phone or email.

"i-BEAT VR" will take place in the next few days at the University of Fribourg and will last about 2.5 hours. A total of 60 people will also take part in this study. You will participate in a social situation or a game in virtual reality (VR). We will also measure your heartbeat and eye movements, which is not associated with any known side effects. In the course of this examination, we will also take a photo of you, which will be deleted at the end of the examination.

"i-BEAT Online" will start in about 4 weeks and last about 15 weeks from then on. A total of 120 people will take part in this study. During the first 6 weeks of the study, you will participate in an internet-based therapy. After a 3-week break, you will take part in another 6-week therapy. One of the two therapies focuses on difficulties with eating, while the other therapy focuses on relationships with other people. It is decided at random which of the two therapies will be started (i.e. both options are chosen with a probability of 50%). The treatment is based on a tested therapy programme for adults, which has now been adapted for adolescents/young adults. On the one hand, content is conveyed via the internet, while on the other hand, a weekly exchange with a trained therapist takes place via email.
Alternatively, it is possible to take up treatment at the psychotherapeutic practice centre of the University of Fribourg at regular conditions.

"i-BEAT Epigenetics" can take place as part of the "i-BEAT VR" study, if you take part in it. Then we will ask you to take a swab from your cheek mucosa with a cotton swab. If you do not take part in "i-BEAT VR", we will send you the cotton swabs by post and ask you to send them back to us after the swab has been taken. A total of 60 people will also take part in this study. The investigation of these epigenetic processes does not allow any conclusions to be drawn about hereditary diseases, but we are investigating how environmental influences affect the hereditary dispositions, i.e. the reading of the genes.

In addition, we will ask you in about a year's time to fill out the questionnaire on the internet again. This way we can better understand how your situation has changed during this time.

We conduct this study in accordance with the laws in Switzerland. We also comply with all internationally recognised guidelines. The responsible cantonal ethics committee has reviewed and approved the study. You can also find a description of this study on the website of the Federal Office of Public Health: www.kofam.ch.

4. Procedure

After you have given your written consent to participate in the study, you will be given an appointment for a one-hour diagnostic interview over the phone. This interview will allow us to check whether you meet the criteria for participation in the study and whether the treatment programme is suitable for you. If so, we will invite you to participate voluntarily in one or more of the studies mentioned at the University of Fribourg, Department of Psychology[1].

Your contact person in Fribourg, who will accompany you through the experimental investigations and take the buccal swabs, is a student in clinical psychology and psychotherapy who is trained by the study director.
The treatment persons are trained psychologists at the Centre for Psychotherapy of the University of Fribourg and psychotherapists in advanced training who regularly consult with a psychotherapist certified by the Federal Office of Public Health (FOPH). The treatment persons are supported by Master's students from the Department of Clinical Psychology and Psychotherapy at the University of Fribourg. It will always be the same person who accompanies you through the entire treatment.

You are free to discontinue your participation in the study at any time without any disadvantages for you. In rare cases, we may decide to exclude you from the study. This can happen if, in the course of the programme, we come to the conclusion that it would be better for you to take another or additional treatment in order to

[1] Department of Psychology, University of Fribourg, Rue Faucigny 2, 1700 Fribourg.

effectively reduce your burden. In this case, we would recommend a different or additional treatment programme. If you are taking another treatment in addition to our programme, this is not necessarily a criterion for exclusion.

5. Benefit

The treatment programme in the i-BEAT Online study is based on a proven treatment approach that has been adapted for adolescents/young adults. The other questionnaire studies and laboratory studies are designed to understand how the experience of social exclusion, mood and loss of control over eating are related. In this regard, there is no immediate benefit or harm to be expected from your participation.

6. Right

You are participating in this study voluntarily. If you do not want to take part or want to withdraw your participation later, you do not have to justify this. Moreover, participation in the experimental studies is not a prerequisite for participation in the treatment programme. Your psychological support is guaranteed regardless of your decision. You may ask questions about your participation in the study at any time. Please contact the persons named at the end of this information. Since the interview and the questionnaires are used to check the criteria for participation in the study, we may have to exclude you from the study if you do not meet the participation criteria. If the interview indicates that you have other mental health problems besides binge eating disorder that need treatment, you will be informed of this, subject to your consent, and we will provide you with information about further treatment options. The study staff are subject to a duty of confidentiality towards third parties. This means that no information about the results of the interview will be passed on without your consent. Only in cases of acute danger to yourself (e.g. acute suicidal tendencies) or others will this confidentiality obligation be lifted and you and your relatives will be informed by us and included in the emergency procedure (see Risks and burdens for participants).

7. Duties

As a participant, it is necessary that you adhere to the guidelines and requirements of this study (including completing the sessions according to the schedule, regular contact with the treatment person). The implementation of the therapy programme requires that you are in a sober state (not under the influence of psychoactive substances such as drugs or alcohol) and that you can devote yourself undisturbed to the content of the sessions during the email-based sessions in a quiet place. You agree to report and inform us of new symptoms, new complaints and changes in your condition if you are receiving other treatments and therapies at the same time.

8. Risks and burdens for the participants

For you as a participant in this study, there is no risk and no undesirable side effects are to be expected. In the course of this treatment, you will intensively deal with yourself and various problem areas, which can be a temporary burden. In addition, you will be continuously accompanied by a treatment person. In acutely stressful situations, an emergency procedure will be discussed and initiated. Information on crisis situations and behaviour as well as emergency contact details will be handed out to you at the time of the psycho-diagnostic interview.

When entering the virtual world with VR goggles in one of the experimental examinations, you may temporarily experience so-called motion sickness, the typical symptoms of which are dizziness or general malaise. If you experience any symptoms of motion sickness, we will immediately stop the examination in VR. You can also remove the VR glasses yourself at any time.

For adolescents/young adults of childbearing age: There are no risks and restrictions for adolescents/young adults of childbearing age. However, as pregnancy is associated with changes in eating habits, nutrition and body weight, these changes may affect the effectiveness of the programme. For this reason, if you are currently pregnant, we ask you to postpone your participation until 6 months after the birth.

9. Other treatment options

Apart from the treatment offered and to be reviewed in the study, no other therapy option is offered in the study. However, it is possible to participate in individual psychotherapy for the treatment of binge-eating disorder (binge eating disorder) at the Psychotherapeutic Practice Centre of the University of Fribourg

(https://www3.unifr.ch/psycho/de/psychotherapie) instead of participating in the present treatment study (subject to a fee).

10. Results from the study

The study management will inform you during the study about any new findings that may affect the benefit of the study or your safety and therefore your consent to participate in the study. You will receive this information verbally and in writing.

11. Confidentiality of data and samples

Your personal, psychological and medical data will be collected for this study. Very few professionals will see your unencrypted data, and only to perform tasks within the study. When collecting data for study purposes, the data will be encrypted (coded). Encryption means that all personal data that could identify you (name, date of birth) are deleted and replaced by a key/code. The key list always remains in the institution (University of Fribourg). People who do not know the key cannot therefore draw any conclusions about you. If the results of the study are published, they will not reveal any information about you. Your name will never appear on the internet or in a publication. The individual data (so-called raw data) are made publicly accessible on a digital database so that they can be used for future research purposes. However, this data is always encrypted and can be traced back to you as an individual. All persons who have access to your data within the framework of the study are subject to a duty of confidentiality. The data protection regulations are adhered to and you, as a participant, have the right to view your data at any time.
The buccal swabs are sent in encrypted form to a database/biobank in Bochum, Germany, where they are examined for this project and then immediately destroyed. The key list remains in the institution (University of Fribourg) and only the study management and the treatment staff have access to it. The study management is responsible for ensuring that the same standards are observed abroad as in Switzerland. The competent ethics committee may be given access to the data in order to check data security. The data may also be checked by the institution that initiated the study. The study director may have to disclose your personal data for such checks. All persons must maintain absolute confidentiality.

12. Resignation

You can stop and withdraw from the study at any time if you wish. The data and samples collected up to that point will continue to be evaluated in encrypted form, as information from people who do not want to or cannot complete the treatment is just as important for improving the treatment offered. After the evaluation, your data will be completely encrypted, i.e. your key allocation will be destroyed, so that no one can find out afterwards that the data originally came from you.

13. Compensation for participants

If you are not studying psychology at the University of Fribourg/Freiburg, you will be compensated in the following way. For participation in the study "i-BEAT App" you will receive 100 CHF, and for participation in the study "i-BEAT VR" including participation in the study "i-BEAT Epigenetics" you will receive 150 CHF. The participation in the questionnaire, which you have just filled in, will not be reimbursed. If you are studying psychology at the University of Fribourg/Freiburg, you will receive subject hours for your participation in the studies "i-BEAT App", "i-BEAT VR" and "i-BEAT Epigenetics". Please take into account that students of psychology can compensate required subject hours by substitute performances (see Bachelor plan, page 17, point 9 : Experimental self-awareness www3.unifr.ch/psycho/en/assets/public/bachelor/plans/PLE_Psycho_BA180_De_rat29012019v03.pdf).
Participation in the treatment programme "i-BEAT Online" is free of charge. Expenses such as travel costs, which are only due to participation, will be reimbursed to you in addition. Participation in the study does not entitle you to any commercial developments (e.g. patents).

14. Liability

The University of Fribourg and thus the project management by Prof. Simone Munsch, who initiated the study and is responsible for its implementation, is liable for any damage that might occur to you in connection with the research.
The requirements and procedure for this are regulated by law. For this purpose, the University of Fribourg has taken out insurance with the Basler Versicherungen in order to be able to cover liability in the event of a possible claim during the implementation of the experimental studies or the treatment programme.

If you suffer any damage, you are asked to contact the director of studies, Prof. Simone Munsch.

15. Funding of the study

The study is funded by the Swiss National Science Foundation (SNF 10001C_185387 / 1).

16. Contact person(s)

If you have any questions, uncertainties or emergencies that arise during or after the study, you can contact one of these contact persons at any time:

Prof. Dr. Simone Munsch
Chair of Clinical Psychology and Psychotherapy
Department of Psychology
University of Fribourg
Rue de Faucigny 2
CH-1700 Fribourg
Telephone Prof. Dr. Simone Munsch: + 41 26 300 76 55
Email: simone.munsch@unifr.ch

M.Sc. Felicitas Forrer
Graduate assistant
RM 01 bu. C-2.118
Rue P.A. de Faucigny 2
CH-1700 Fribourg
Tel. +41 26 300 7658
Email: felicitas.forrer@unifr.ch

M.Sc. Verena Müller
SNF doctoral student
RM 01 bu. C-2.118
Rue P.A. de Faucigny 2
CH-1700 Fribourg
Tel. M.Sc. Verena Müller: +41 26 300 7659
Email: verena.mueller@unifr.ch
M.Sc. Adrian Naas
SNSF doctoral student
RM 01 bu. C-2.113
Rue P.A. de Faucigny 2
CH-1700 Fribourg
Tel. M.Sc. Adrian Naas: +41 26 300 7478
Email: adrian.naas@unifr.ch

In psychological emergency situations, you can also dial the 24-hour emergency number of the Swiss worry line (Dargebotene Hand): 143.

17. Glossary (terms requiring explanation):

Binge eating disorder: According to the diagnostic criteria described in the Diagnostic and Statistical Manual of Mental Disorders (DSM-5, APA, 2013), this form of eating disorder is characterised by eating a large amount of food over a defined period of time and losing control over what and how much you eat. These so-called "eating attacks" are often accompanied by eating very quickly, in a jumbled manner, without hunger and to the point of an unpleasant feeling of fullness. As a result of these eating binges, feelings of shame, guilt and dejection set in.
Epigenetics: Field of biology and medicine that deals with which gene becomes active under which environmental influences. Epigenetic findings can, for example, help to understand under which biological and environmental conditions adolescents/young adults are more prone to binge eating.

If you would like to get more information or if other terms seem unclear, please visit our project homepage www.unifr.ch/go/i-BEAT. There we explain, for example, what emotion regulation is, what binge eating disorder (BES) is and how it differs from binge eating and loss of control eating, and what you can do against binge eating.

Declaration of consent

Written declaration of consent to participate in a study project

Please read this form carefully. Please ask if there is anything you do not understand or would like to know. Your written consent is required for participation.

**BASEC number (after submission):**	ID 2019-01277	
**Study title:**	**i-BEAT**<br>Binge-Eating Adolescent and Young Adults Treatment<br>a research and internet-based treatment programme for adolescents and young adults with binge eating **disorder.**	
**responsible institution:**	Prof. Dr. Simone Munsch<br>Chair of Clinical Psychology and Psychotherapy<br>Department of Psychology<br>University of Fribourg, Switzerland	
**Place of implementation**:	University of Fribourg, Switzerland	
**Responsible director of studies at the place of study:**	Prof. Dr. Simone Munsch<br>University of Fribourg, Chair of Clinical Psychology and Psychotherapy<br>Department of Psychology<br>University of Fribourg, Switzerland	
**Participant:**<br>Name and first name in block capitals:<br><br>Date of birth:<br>Email address:<br>Gender:	☐ female	☐ male

- I was informed verbally and in writing by the undersigned study director about the purpose, the procedure of the study, about possible advantages and disadvantages as well as about possible risks.
- I am voluntarily participating in this study and accept the content of the written information provided. I have had sufficient time to make my decision.
- My questions in connection with participation in this study have been answered. I will keep the written information and receive a copy of my written informed consent.
- I have been informed that the psycho-diagnostic interview and questionnaires at the beginning of the study will check the criteria for participation and that I may be excluded from the study if I do not meet the criteria.
- I was informed about possible other treatments and treatment procedures.
- I agree that the responsible experts of the study management and the responsible ethics committee may inspect my unencrypted data for testing and control purposes, but in strict compliance with confidentiality.
- I am informed of study results that directly affect my health. If I do not wish to be informed, I inform the study director.

- I know that my health-related and personal data (and samples) from this study can only be passed on in encrypted form for research purposes, including abroad.
- I can withdraw from the study participation at any time and without giving reasons. My further psychotherapeutic treatment is always guaranteed regardless of the study participation and of the participation in the experimental investigations. The data and samples collected up to the time of withdrawal will be used for the evaluation for the study.
- I am informed that the liability insurance of the University of Fribourg will cover any damages.
- I am aware that I must comply with the obligations stated in the participant information. In the interest of my health, the study management may exclude me from the study at any time.
- I am informed that participation in the experimental examinations in virtual reality and in the laboratory as well as the examination of biological factors are not a prerequisite for participation in the i-BEAT treatment programme.

<u>Please tick:</u>

I am taking part in the following/the following examinations:
☐ i-BEAT App
☐ i-BEAT VR
☐ i-BEAT Online
☐ i-BEAT Epigenetics

☐ In the event of acute danger to self or others, I authorise the study management to release me from the duty of confidentiality towards third parties and to involve my relatives in the emergency procedure.

☐ I would like to be informed about the results of the psychodiagnostic examination.

☐ I do NOT wish to be informed about the results of the psychodiagnostic examination and have been informed that my wish will be respected, except in the case of acute mental conditions that are dangerous to myself or others.

☐ My relatives should in any case be informed about my participation and about the results of the psychodiagnostic examinations.
- Information to my relatives after consultation with me will be in writing: ☐
- Information to my relatives after consultation with me is given verbally: ☐

Contact details of relatives (informing relatives about study participation if desired and in the case of acute danger to self and/or others).

Name / First name<br><br>Address<br><br>Email address<br><br>Telephone number	

Place, date	Signature Participant

Confirmation by the investigator: I hereby confirm that I have explained the nature, significance and scope of the study to this participant. I affirm that I will fulfil all obligations in connection with this study in accordance

with applicable law. If, at any time during the conduct of the study, I learn of any issues that may affect the participant's willingness to participate in the study, I will inform him/her immediately.

☐ The adolescent's/young adult's capacity for judgement in terms of understanding the content of the study and the effort involved in participating has been assessed (summary in the adolescent's/young adult's own words) and is present.

Place, date	Surname and first name of the inspector in block capitals<br><br>Signature of the test person

D4

Short title: i-BEAT - Binge-Eating Adolescent and Young Adults Treatment - a research and internet-based treatment programme for adolescents and young adults with binge eating **disorder.**

Study information for young adult participants without binge eating disorder
Project management: Prof. Dr. Simone Munsch, University of Fribourg, Switzerland

Dear young person

We would like to ask you if you would like to participate in a clinical trial. In the following, the study projects are presented to you.

More detailed information

1. Aim of the studies

There are three studies in this project: "i-BEAT App", "i-BEAT VR" and "i-BEAT Epigenetics". With these we want to investigate different things:
- "i-BEAT App": Here we want to use a smartphone app to learn more about the experience of social exclusion and its impact on mood and eating behaviour in adolescents and young adults with binge eating disorder (BES) and/or regular loss of control and compare this with the experience of adolescents/young adults who do not have such experiences.
- "i-BEAT VR": In this laboratory study, we investigate the psychological and physiological effects of an interpersonal situation in virtual reality (VR).
- "i-BEAT Epigenetics": Here we take a saliva sample through which we can examine how experiences in everyday life can affect the expression of genetic traits.

What all the studies have in common is that we want to use them to better understand how loss of control over eating occurs, what perpetuates this problem and how it can best be treated. While "i-BEAT App" focuses on the personal experience in everyday life, with "i-BEAT VR" and "i-BEAT Epigenetics" we are investigating even more closely how the experience of loss of control when eating is related to physical measures (e.g. the heartbeat) and the expression of genetic characteristics.

2. Selection

This study is open to all adolescents/young adults between the ages of 14 and 24 who suffer from binge eating disorder (BES), regular binge eating and/or a feeling of loss of control over food, as well as people without these characteristics as a comparison group. You will also need a smartphone, internet access to receive emails and a good enough knowledge of German to participate in one of the studies. Adolescents/young adults who also suffer from another serious mental illness that needs to be treated first or adolescents/young adults who are pregnant cannot participate. We will not conduct a pregnancy test, but will rely on the information provided by the participants. Also excluded from participation are adolescents and young adults who are in an employment relationship with Prof. Munsch or the working group of the Chair of Prof. Munsch or who work for Prof. Munsch.

3. General information

This study is being conducted by the University of Fribourg, Switzerland, under the direction of Prof. Dr. Simone Munsch. With the help of the internet questionnaire that you filled out, we were already able to gather initial information about your situation. Next, a more detailed interview will take place by telephone, which will last about an hour. Afterwards, you can take part in one or more of the studies described. Participation in studies takes different lengths of time and takes place at different times.

"i-BEAT App" will last a total of 7 days. A total of 60 people will take part in this study. You will be asked to report on your experiences and how you are feeling 3 times a day with the help of a smartphone app. This will take about 30 minutes each day. This study can start in the next few days. If you take part in the "i-BEAT VR"

study, we can use this opportunity to show you how to use the smartphone app. If you do not take part in i-BEAT VR, we will instruct you about this by phone or email.

"i-BEAT VR" will take place in the next few days at the University of Fribourg and will last about 2.5 hours. A total of 60 people will also take part in this study. You will participate in a social situation or a game in virtual reality (VR). We will also measure your heartbeat and eye movements, which is not associated with any known side effects. In the course of this examination, we will also take a photo of you, which will be deleted at the end of the examination.

"i-BEAT Epigenetics" can take place as part of the "i-BEAT VR" study, if you take part in it. Then we will ask you to take a swab from your cheek mucosa with a cotton swab. If you do not take part in "i-BEAT VR", we will send you cotton swabs by post and ask you to send them back to us after the swab has been taken. A total of 60 people will also take part in this study. The investigation of these epigenetic processes does not allow any conclusions to be drawn about hereditary diseases, but we are investigating how environmental influences affect the hereditary dispositions, i.e. the reading of the genes.

In addition, we will ask you in about a year's time to fill out the questionnaire on the internet again. This way we can better understand how your situation has changed during this time.

We conduct this study in accordance with the laws in Switzerland. We also comply with all internationally recognised guidelines. The responsible cantonal ethics committee has reviewed and approved the study. You can also find a description of this study on the website of the Federal Office of Public Health: www.kofam.ch.

4. Procedure

After you have given your written consent to participate in the study, you will be given an appointment for a one-hour diagnostic interview over the phone. This interview will allow us to check whether you meet the criteria for participation in the study and whether the treatment programme is suitable for you. If so, we will invite you to participate voluntarily in one or more of the studies mentioned at the University of Fribourg, Department of Psychology[1].

Your contact person in Fribourg, who will accompany you through the experimental examinations and take the buccal swabs, is a student of clinical psychology and psychotherapy who is trained by the study director.

You are free to discontinue your participation in the study at any time without any disadvantages for you. In rare cases, we may decide to exclude you from the study. This can happen if, in the course of the programme, we come to the conclusion that participation is too much of a burden for you or if you repeatedly fail to keep the agreed appointments.

5. Benefit

The studies are designed to understand how the experience of social exclusion, mood and loss of control over eating are related. You are not expected to gain any immediate benefit from participating, but you are not expected to suffer any harm or disadvantage.

6. Right

You are participating in this study voluntarily. If you do not want to take part or later withdraw your participation, you do not have to justify this. However, you will only be paid for participating in a sub-study if you complete it in full. You can ask questions about your participation in the study at any time. Please contact the persons named at the end of this information. As the interview and questionnaires are used to check the criteria for participation in the study, we may have to exclude you from the study if you do not meet the participation criteria. If the interview indicates that you have mental health problems that require treatment, you will be informed of this, subject to your consent, and you will receive information from us about further treatment options. The study staff are bound by the duty of confidentiality towards third parties, including your parents/guardians. This means that no information about the results of the interview will be passed on without

[1] Department of Psychology, University of Fribourg, Rue Faucigny 2, 1700 Fribourg.

your consent. Only in the case of acute danger to yourself (e.g. acute suicidal tendencies) or others will this confidentiality obligation be lifted and you and your parents/guardians will be informed by us and included in the emergency procedure (see Risks and burdens for participants).

7. Duties

As a participant, it is necessary that you adhere to the guidelines and requirements of the individual sub-studies (including completing the examinations in the laboratory, filling out the questionnaires, answering the questions in the "i-BEAT App" study, regular contact with the study coordination). You are obliged to report any changes in your condition.

8. Risks and burdens for the participants

For you as a participant in this study, there is no risk and no undesirable side effects are to be expected. We will accompany and support you in stressful situations during the study. Information on crisis situations and behaviour as well as emergency contact details will be handed out to you at the time of the psycho-diagnostic interview.

When entering the virtual world with VR goggles in one of the experimental examinations, you may temporarily experience so-called motion sickness, the typical symptoms of which are dizziness or general malaise. If you experience any symptoms of motion sickness, we will immediately stop the examination in VR. You can also remove the VR glasses yourself at any time.

For adolescents/young adults of childbearing age: There are no risks and limitations for adolescents/young adults of childbearing age. However, as pregnancy is associated with changes in eating habits, diet and body weight, these changes may influence the study results. For this reason, we ask you to postpone your participation until 6 months after birth if you are currently pregnant.

9. Results from the study

The study management will inform you during the study about any new findings that may affect the benefit of the study or your safety and therefore your consent to participate in the study. You will receive this information verbally and in writing.

10. Confidentiality of data and samples

Your personal, psychological and medical data will be collected for this study. Very few professionals will see your unencrypted data, and only to perform tasks within the study. When collecting data for study purposes, the data will be encrypted (coded). Encryption means that all personal data that could identify you (name, date of birth) are deleted and replaced by a key/code. The key list always remains in the institution (University of Fribourg). People who do not know the key cannot therefore draw any conclusions about you. If the results of the study are published, there will be no reference to you. Your name will never appear on the internet or in a publication. The individual data (so-called raw data) are made publicly accessible on a digital database so that they can be used for future research purposes. However, this data is always encrypted and can never be traced back to you as an individual. All persons who have access to your data in the course of the study are bound to professional secrecy. The data protection regulations are adhered to and you, as a participant, have the right to view your data at any time.

The buccal swabs are sent in encrypted form to a database/biobank in Bochum, Germany, where they are examined for this project and then immediately destroyed. The key list remains in the institution (University of Fribourg) and only the study management and the treatment staff have access to it. The study management is responsible for ensuring that the same standards are observed abroad as in Switzerland. The competent ethics committee may be given access to the data in order to check data security. The data may also be checked by the institution that initiated the study. The study director may have to disclose your personal data for such checks. All persons must maintain absolute confidentiality.

11. Resignation

You can stop and withdraw from the study at any time if you wish. The data and samples collected up to that point will continue to be evaluated in encrypted form, as information from people who do not want to or cannot complete the treatment is just as important for improving the treatment offered. After the evaluation, your data will be completely encrypted, i.e. your key allocation will be destroyed, so that no one can find out afterwards that the data originally came from you.

12. Compensation for participants

If you are not studying psychology at the University of Fribourg/Freiburg, you will be compensated in the following way. You will receive CHF 100 for participating in the "i-BEAT App" study, and CHF 150 for participating in the "i-BEAT VR" study, including participation in the "i-BEAT Epigenetics" study. If you are studying psychology at the University of Fribourg/Freiburg, you will receive subject hours for your participation in the "i-BEAT App", "i-BEAT VR" and "i-BEAT Epigenetics" studies. Please take into account that psychology students can compensate for required subject hours by substitute work (see Bachelor Plan, page 17, point 9: Experimental self-awareness www3.unifr.ch/psycho/en/assets/public/bachelor/plans/PLE_Psycho_BA180_De_rat29012019v03.pdf). You will also be reimbursed for expenses such as travel expenses that are only incurred as a result of participation. Participation in the study does not entitle you to any commercial developments (e.g. patents).

13. Liability

The University of Fribourg and thus the project management by Prof. Simone Munsch, who initiated the study and is responsible for its implementation, is liable for any damage that might occur to you in connection with the research. The conditions and the procedure for this are regulated by law. For this purpose, the University of Fribourg has taken out insurance with the Basler Versicherungen in order to be able to cover liability in the event of any damage occurring during the performance of the experimental studies or the treatment programme. If you suffer any damage, you are requested to contact the head of the study, Prof. Simone Munsch.

14. Funding of the study

The study is funded by the Swiss National Science Foundation (SNF 10001C_185387 / 1).

15. Contact person(s)

If you have any questions, uncertainties or emergencies that arise during or after the study, you can contact one of these contact persons at any time:

Prof. Dr. Simone Munsch
Chair of Clinical Psychology and Psychotherapy
Department of Psychology, University of Fribourg
Rue de Faucigny 2
CH-1700 Fribourg
Telephone Prof. Dr. Simone Munsch: + 41 26 300 76 55
Email: simone.munsch@unifr.ch
Felicitas Forrer
Graduate assistant
RM 01 bu. C-2.118
Rue P.A. de Faucigny 2
CH-1700 Fribourg
Tel. +41 26 300 7658
Email: felicitas.forrer@unifr.ch

M.Sc. Verena Müller
SNF doctoral student
RM 01 bu. C-2.118
Rue P.A. de Faucigny 2
CH-1700 Fribourg
Tel. M.Sc. Verena Müller: +41 26 300 7659
Email: verena.mueller@unifr.ch

M.Sc. Adrian Naas
SNSF doctoral student
RM 01 bu. C-2.113

Rue P.A. de Faucigny 2
CH-1700 Fribourg
Tel. M.Sc. Adrian Naas: +41 26 300 7478
Email: adrian.naas@unifr.ch

In psychological emergency situations, you can also dial the 24-hour emergency number of the Swiss worry line (Dargebotene Hand): 143.

16. Glossary (terms requiring explanation):

Binge eating disorder: According to diagnostic criteria described in the Diagnostic and Statistical Manual of Mental Disorders (DSM-5, APA, 2013), this form of eating disorder is characterised by eating a large amount of food in a definable period of time and losing control over what and how much they eat. These so-called "eating attacks" are often accompanied by eating very quickly, in a jumbled manner, without hunger and to the point of an unpleasant feeling of fullness. As a result of these eating binges, feelings of shame, guilt and dejection set in.

Epigenetics: Field of biology and medicine that deals with which gene becomes active under which environmental influences. Epigenetic findings can, for example, help to understand under which biological and environmental conditions adolescents/young adults are more prone to binge eating.

Declaration of consent

Written declaration of consent to participate in a study project

Please read this form carefully. Please ask if there is anything you do not understand or would like to know. Your written consent is required for participation.

**BASEC number (after submission):**	ID 2019-01277
**Study title:**	**i-BEATBinge-Eating** Adolescent and Young Adults Treatment a research and internet-based treatment programme for adolescents and young adults with binge eating **disorder.**
**responsible institution:**	Prof. Dr. Simone Munsch<br>Chair of Clinical Psychology and Psychotherapy<br>Department of Psychology<br>University of Fribourg, Switzerland
**Place of implementation**:	University of Fribourg, Switzerland
**Responsible director of studies at the place of study:**	Prof. Dr. Simone Munsch<br>University of Fribourg, Chair of Clinical Psychology and Psychotherapy<br>Department of Psychology<br>University of Fribourg, Switzerland
**Participant:**<br>Name and first name in block capitals:<br><br>Date of birth:<br>Email address:<br>Gender:	 <br><br><br><br>☐ female ☐ male

- I have been informed verbally and in writing by the undersigned study director about the purpose, the procedure of the study, about possible advantages and disadvantages as well as about possible risks.
- I am voluntarily participating in this study and accept the content of the written information provided. I have had sufficient time to make my decision.
- My questions in connection with participation in this study have been answered. I will keep the written information and receive a copy of my written informed consent.
- I have been informed that the psycho-diagnostic interview and questionnaires at the beginning of the study will check the criteria for participation and that I may be excluded from the study if I do not meet the criteria.
- I was informed about possible other treatments and treatment procedures.
- I agree that the responsible experts of the study management and the responsible ethics committee may inspect my unencrypted data for testing and control purposes, but in strict compliance with confidentiality.
- I am informed of study results that directly affect my health. If I do not wish to be informed, I inform the study director.
- I know that my health-related and personal data (and samples) from this study can only be passed on in encrypted form for research purposes, including abroad.
- I can withdraw from the study participation at any time and without giving reasons. My further psychotherapeutic treatment is always guaranteed regardless of the study participation and of the participation in the experimental investigations. The data and samples collected up to the time of withdrawal will be used for the evaluation for the study.
- I am informed that the liability insurance of the University of Fribourg will cover any damages.
- I am aware that I must comply with the obligations stated in the participant information. In the interest of my health, the study management may exclude me from the study at any time.

Please tick:

I am taking part in the following/the following examinations:
☐ i-BEAT App
☐ i-BEAT VR
☐ i-BEAT Epigenetics

☐ I would like to be informed if psychological stress is detected during psychodiagnostics. In addition, I have been informed that in the event of acute mental states that are dangerous to myself or others, my parents/guardians will be informed and included in the emergency procedure.

☐ I do NOT wish to be informed about the results of the psychodiagnostic examination and have been informed that my wish will be respected, except in the case of acute mental conditions that are dangerous to myself or others.

☐ My parents/guardians should be informed about my participation and about the results of the psycho-diagnostic examinations in any case.
- Information to my parents after consultation with me will be in writing: ☐
- Information to my parents after consultation with me is verbal: ☐

Contact details of parents/guardians (informing parents about study participation if desired and in case of acute danger to self and/or others).

Name / First name<br><br>Address<br><br>Email address<br><br>Telephone number	

Place, date	Signature Participant

Confirmation by the investigator: I hereby confirm that I have explained the nature, significance and scope of the study to this participant. I affirm that I will fulfil all obligations related to this study in accordance with applicable law. If at any time during the conduct of the study I become aware of any issues that may affect the participant's willingness to participate in the study, I will inform him/her immediately.

☐ The young person's capacity for judgement in terms of understanding the content of the study and the effort involved in participating has been assessed (summary in the young person's own words) and is present.

Place, date	Surname and first name of the inspector in block capitals<br><br>Signature of the test person

UNIVERSITY OF FRIBOURG SWITZERLAND
DEPARTMENT OF PSYCHOLOGY
PROF. DR. SIMONE MUNSCH

UNIVERSITÉ DE FRIBOURG
UNIVERSITÄT FREIBURG

D5

Short title: i-BEAT - Binge-Eating Adolescent and Young Adults Treatment - a research and internet-based treatment programme for adolescents and young adults with binge eating **disorder.**

Study information for young adult participants without binge eating disorder

Project management: Prof. Dr. Simone Munsch, University of Fribourg, Switzerland

Dear participant,

We would like to ask you if you would like to participate in a clinical trial. In the following, the study project is presented to you.

More detailed information

1. Aim of the studies

There are three studies in this project: "i-Beat App", "i-Beat VR" and "i-Beat Epigenetics". With these we want to investigate different things:
- "i-Beat App": here we would like to learn more about the experience of social exclusion and its effects on mood and eating behaviour in adolescents and young adults with binge eating disorder (BES: binge eating disorder) and/or regular loss of control and compare this with the experience of adolescents/young adults who do not have such experiences.
- "i-Beat VR": in this laboratory study, we investigate the psychological and physiological effects of an interpersonal situation in virtual reality (VR).
- "i-BEAT Epigenetics": here we take a saliva sample through which we can examine how experiences in everyday life can affect the expression of genetic traits.

What all the studies have in common is that we want to use them to better understand how loss of control over eating arises, what perpetuates this problem and how it can best be treated. While "i-Beat App" focuses on the personal experience in everyday life, with "i-Beat VR" and "i-Beat Epigenetics" we are investigating even more closely how the experience of loss of control when eating is related to physical measures (e.g. the heartbeat) and genetics. With "i-Beat online" we are trying to better understand how to optimally treat the issue.

2. Selection

This study is open to all adolescents/young adults between the ages of 14 and 24 who suffer from binge eating disorder (BES), regular binge eating and/or a feeling of loss of control over food, as well as people without these characteristics as a comparison group. You will also need a smartphone, internet access to receive emails and a good enough knowledge of German to participate in one of the studies. Adolescents/young adults who also suffer from another serious mental illness that needs to be treated first or adolescents/young adults who are pregnant cannot participate. We will not conduct a pregnancy test, but will rely on the information provided by the participants. Also excluded from participation are adolescents and young adults who are in an employment relationship with Prof. Munsch or the working group of the Chair of Prof. Munsch or who work for Prof. Munsch.

3. General information

This study is being conducted by the University of Fribourg, Switzerland, under the direction of Prof. Dr. Simone Munsch. With the help of the internet questionnaire that you filled out, we were already able to gather initial information about your situation. Next, a more detailed interview will take place by telephone, which will last about an hour. Afterwards, you can take part in one or more of the studies described. Participation in studies takes different lengths of time and takes place at different times.

"**i-Beat App**" will last a total of 7 days. A total of 60 people will take part in this study. You will be asked to report on your experiences and how you feel 3 times a day with the help of a smartphone app. This will take about 30 minutes each day. This study can start in the next few days. If you take part in the "i-Beat VR" study, we can use this opportunity to show you how to use the smartphone app. If you are not participating in i-Beat VR, we will instruct you about this by phone or email.

"**i-Beat VR**" will take place at the University of Fribourg in the next few days and will last about 2.5 hours. A total of 60 people will also take part in this study. You will participate in a social situation or a game in virtual reality (VR). We will also measure your heartbeat and eye movements, which is not associated with any known side effects. In the course of this examination, we will also take a photo of you, which will be deleted at the end of the examination.

"**i-Beat Epigenetics**" can take place as part of the "i-Beat VR" study, if you take part in it. Then we will ask you to take a swab from your cheek mucosa with a cotton swab. If you do not participate in "i-Beat VR", we will send you the cotton swabs by post and ask you to send them back to us after the swab has been taken. A total of 60 people will also take part in this study. The investigation of these epigenetic processes does not allow any conclusions to be drawn about hereditary diseases, but we are investigating how environmental influences affect the genetic make-up, i.e. the reading of the genes.

In addition, we will ask you in about a year's time to fill out the **questionnaire** on the internet again. This way we can better understand how your situation has changed during this time.

We conduct this study in accordance with the laws in Switzerland. We also comply with all internationally recognised guidelines. The responsible cantonal ethics committee has reviewed and approved the study. You can also find a description of this study on the website of the Federal Office of Public Health: www.kofam.ch.

4. Procedure

After you have given your written consent to participate in the study, you will be given an appointment for a one-hour diagnostic interview over the phone. This interview will allow us to check whether you meet the criteria for participation in the study and whether the treatment programme is suitable for you. If so, we will invite you to participate voluntarily in one or more of the studies mentioned above at the University of Fribourg, Department of Psychology[1].

Your contact person in Fribourg, who will accompany you through the experimental examinations and take the cheek swabs, is a student in clinical psychology and psychotherapy who is trained by the study director. You are free to discontinue your participation in the study at any time without any disadvantage to you. In rare cases, we may decide to exclude you from the study. This can happen if, in the course of the programme, we come to the conclusion that you are too burdened by your participation or if you repeatedly fail to keep the agreed appointments.

5. Benefit

The studies are designed to understand how the experience of social exclusion, mood and loss of control over eating are related. You are not expected to gain any immediate benefit from participating, but you are not expected to suffer any harm or disadvantage.

6. Right

You are participating in this study voluntarily. If you do not want to participate or withdraw your participation later, you do not have to justify this. However, you will only be paid for participating in a sub-study if you complete it in full. You can ask questions about your participation in the study at any time. Please contact the persons named at the end of this information. As the interview and questionnaires are used to check the criteria for participation in the study, we may have to exclude you from the study if you do not meet the participation criteria. If the interview indicates that you have mental health problems that require treatment, you will be

[1] Department of Psychology, University of Fribourg, Rue Faucigny 2, 1700 Fribourg.

informed of this, subject to your consent, and we will provide you with information about further treatment options. The study staff are subject to a duty of confidentiality towards third parties. This means that no information about the results of the interview will be passed on without your consent. Only in the case of acute danger to yourself (e.g. acute suicidal tendencies) or others will this confidentiality obligation be lifted and you and your relatives will be informed by us and included in the emergency procedure (see Risks and burdens for participants).

7. Duties

As a participant, it is necessary that you adhere to the guidelines and requirements of the individual sub-studies (including completing the examinations in the laboratory, filling out the questionnaires, answering the questions in the "i-BEAT App" study, regular contact with the study coordination). You are obliged to report any changes in your condition.

8. Risks and burdens for the participants

For you as a participant in this study, there is no risk and no undesirable side effects are to be expected. We will accompany and support you in stressful situations during the study. In the event of acutely stressful situations, an emergency procedure will be discussed and initiated. Information on crisis situations and behaviour as well as emergency contact details will be given to you at the time of the psycho-diagnostic interview.
When entering the virtual world with VR goggles in one of the experimental examinations, you may temporarily experience so-called motion sickness, the typical symptoms of which are dizziness or general malaise. If you experience any symptoms of motion sickness, we will immediately stop the examination in VR. You can also remove the VR glasses yourself at any time.
For adolescents/young adults of childbearing age: There are no risks and limitations for adolescents/young adults of childbearing age. However, as pregnancy is associated with changes in eating habits, diet and body weight, these changes may influence the study results. For this reason, we ask you to postpone your participation until 6 months after birth if you are currently pregnant.

9. Results from the study

The study management will inform you during the study about any new findings that may affect the benefit of the study or your safety and therefore your consent to participate in the study. You will receive this information verbally and in writing.

10. Confidentiality of data and samples

Your personal, psychological and medical data will be collected for this study. Very few professionals will see your unencrypted data, and only to perform tasks within the study. When collecting data for study purposes, the data will be encrypted (coded). Encryption means that all personal data that could identify you (name, date of birth) are deleted and replaced by a key/code. The key list always remains in the institution (University of Fribourg). People who do not know the key cannot therefore draw any conclusions about you. If the results of the study are published, there will be no reference to you. Your name will never appear on the internet or in a publication. The individual data (so-called raw data) are made publicly accessible on a digital database so that they can be used for future research purposes. However, this data is always encrypted and can never be traced back to you as an individual. All persons who have access to your data in the course of the study are bound to professional secrecy. The data protection regulations are adhered to and you, as a participant, have the right to view your data at any time.
The buccal swabs are sent in encrypted form to a database/biobank in Bochum, Germany, where they are examined for this project and then immediately destroyed. The key list remains in the institution (University of Fribourg) and only the study management and the treatment staff have access to it. The study management is responsible for ensuring that the same standards are observed abroad as in Switzerland. The competent ethics committee may be given access to the data in order to check data security. The data may also be checked by the institution that initiated the study. The study director may have to disclose your personal data for such checks. All persons must maintain absolute confidentiality.

11. Resignation

You can stop and withdraw from the study at any time if you wish. The data and samples collected up to that point will continue to be evaluated in encrypted form, as information from people who do not want to or cannot complete the treatment is just as important for improving the treatment offered. After the evaluation, your data will be completely encrypted, i.e. your key allocation will be destroyed, so that no one can find out afterwards that the data originally came from you.

12. Compensation for participants

If you are not studying psychology at the University of Fribourg/Freiburg, you will be compensated in the following way. You will receive CHF 100 for participating in the "i-BEAT App" study, and CHF 150 for participating in the "i-BEAT VR" study, including participation in the "i-BEAT Epigenetics" study. If you are studying psychology at the University of Fribourg/Freiburg, you will receive subject hours for your participation in the studies "i-BEAT App", "i-BEAT VR" and "i-BEAT Epigenetics". Please take into account that psychology students can compensate for required subject hours by substitute work (see Bachelor Plan, page 17, point 9: Experimental self-awareness www3.unifr.ch/psycho/en/assets/public/bachelor/plans/PLE_Psycho_BA180_De_rat29012019v03.pdf). You will also be reimbursed for expenses such as travel expenses that are only incurred as a result of participation. Participation in the study does not entitle you to any commercial developments (e.g. patents).

13. Liability

The University of Fribourg and thus the project management by Prof. Simone Munsch, who initiated the study and is responsible for its implementation, is liable for any damage that might occur to you in connection with the research.

The requirements and procedure for this are regulated by law. For this purpose, the University of Fribourg has taken out insurance with the Basler Versicherungen in order to be able to cover liability in the event of a possible claim during the implementation of the experimental studies or the treatment programme.

If you suffer any damage, please contact the director of studies, Prof. Simone Munsch.

14. Funding of the study

The study is funded by the Swiss National Science Foundation (SNF 10001C_185387 / 1).

15. Contact person(s)

If you have any questions, uncertainties or emergencies that arise during or after the study, you can contact one of these contact persons at any time:

Prof. Dr. Simone Munsch
Chair of Clinical Psychology and Psychotherapy
Department of Psychology
University of Fribourg
Rue de Faucigny 2
CH-1700 Fribourg
Telephone Prof. Dr. Simone Munsch: + 41 26 300 76 55
Email: simone.munsch@unifr.ch

M.Sc. Felicitas Forrer
Graduate assistant
RM 01 bu. C-2.118
Rue P.A. de Faucigny 2
CH-1700 Fribourg
Tel. +41 26 300 7658
Email: felicitas.forrer@unifr.ch

M.Sc. Verena Müller

SNF doctoral student
RM 01 bu. C-2.118
Rue P.A. de Faucigny 2
CH-1700 Fribourg
Tel. M.Sc. Verena Müller: +41 26 300 7659
Email: verena.mueller@unifr.ch

M.Sc. Adrian Naas
SNSF doctoral student
RM 01 bu. C-2.113
Rue P.A. de Faucigny 2
CH-1700 Fribourg
Tel. M.Sc. Adrian Naas: +41 26 300 7478
Email: adrian.naas@unifr.ch

In psychological emergency situations, you can also dial the 24-hour emergency number of the Swiss worry line (Dargebotene Hand): 143.

16. Glossary (terms requiring explanation):

Binge eating disorder: According to diagnostic criteria described in the Diagnostic and Statistical Manual of Mental Disorders (DSM-5, APA, 2013), this form of eating disorder is characterised by eating a large amount of food in a definable period of time and losing control over what and how much they eat. These so-called "eating attacks" are often accompanied by eating very quickly, in a jumbled manner, without hunger and to the point of an unpleasant feeling of fullness. As a result of these eating binges, feelings of shame, guilt and dejection set in.

Epigenetics: Field of biology and medicine that deals with which gene becomes active under which environmental influences. Epigenetic findings can, for example, help to understand under which biological and environmental conditions adolescents/young adults are more prone to binge eating.

Declaration of consent

Written declaration of consent to participate in a study project

Please read this form carefully. Please ask if there is anything you do not understand or would like to know. Your written consent is required for participation.

**BASEC number (after submission):**	ID 2019-01277
**Study title:**	**i-BEATBinge-Eating** Adolescent and Young Adults Treatment a research and internet-based treatment programme for adolescents and young adults with binge eating **disorder.**
**responsible institution:**	Prof. Dr. Simone Munsch<br>Chair of Clinical Psychology and Psychotherapy<br>Department of Psychology<br>University of Fribourg, Switzerland
**Place of implementation**:	University of Fribourg, Switzerland
**Responsible director of studies at the place of study:**	Prof. Dr. Simone Munsch<br>University of Fribourg, Chair of Clinical Psychology and Psychotherapy

	Department of Psychology<br>University of Fribourg, Switzerland
**Participant:**<br>Name and first name in block capitals:	
Date of birth:	
Email address:	
Gender:	☐ female ☐ male

- I have been informed verbally and in writing by the undersigned study director about the purpose, the procedure of the study, about possible advantages and disadvantages as well as about possible risks.
- I am voluntarily participating in this study and accept the content of the written information provided. I have had sufficient time to make my decision.
- My questions in connection with participation in this study have been answered. I will keep the written information and receive a copy of my written informed consent.
- I have been informed that the psycho-diagnostic interview and questionnaires at the beginning of the study will check the criteria for participation and that I may be excluded from the study if I do not meet the criteria.
- I was informed about possible other treatments and treatment procedures.
- I agree that the responsible experts of the study management and the responsible ethics committee may inspect my unencrypted data for testing and control purposes, but in strict compliance with confidentiality.
- I am informed of study results that directly affect my health. If I do not wish to be informed, I inform the study director.
- I know that my health-related and personal data (and samples) from this study can only be passed on in encrypted form for research purposes, including abroad.

- I can withdraw from the study participation at any time and without giving reasons. My further psychotherapeutic treatment is always guaranteed regardless of the study participation and of the participation in the experimental investigations. The data and samples collected up to the time of withdrawal will be used for the evaluation for the study.
- I am informed that the liability insurance of the University of Fribourg will cover any damages.
- I am aware that the obligations stated in the participant information must be complied with. In the interest of my health, the study management may exclude me from the study at any time.

<u>Please tick:</u>

I am taking part in the following/the following examinations:
☐ i-Beat App
☐ i-Beat VR
☐ i-Beat Epigenetics

☐ I would like to be informed if psychological stress is detected during psychodiagnostics. In the event of acute danger to self or others, I authorise the study management to release me from the duty of confidentiality towards third parties and to involve my relatives in the emergency procedure.

☐ I would like to be informed about the results of the psychodiagnostic examination.

☐ I do NOT wish to be informed about the results of the psychodiagnostic examination and have been informed that my wish will be respected, except in the case of acute mental conditions that are dangerous to myself or others.

☐ My relatives should in any case be informed about my participation and about the results of the psychodiagnostic examinations.

- Information to my relatives after consultation with me will be in writing: ☐
- Information to my relatives after consultation with me is given verbally: ☐

Contact details of relatives (informing relatives about study participation if desired and in the case of acute danger to self and/or others).

Name / First name<br><br>Address<br><br>Email address<br><br>Telephone number	

Place, date	Signature Participant

Confirmation by the investigator: I hereby confirm that I have explained the nature, significance and scope of the study to this participant. I affirm that I will fulfil all obligations in connection with this study in accordance with applicable law. If, at any time during the conduct of the study, I learn of any issues that may affect the participant's willingness to participate in the study, I will inform him/her immediately.

☐ The adolescent's/young adult's capacity for judgement in terms of understanding the content of the study and the effort involved in participating has been assessed (summary in the adolescent's/young adult's own words) and is present.

Place, date	Surname and first name of the inspector in block capitals<br><br>Signature of the test person

D6

Short title: i-BEAT - Binge-Eating Adolescent and Young Adults Treatment - a research and internet-based treatment programme for adolescents and young adults with binge eating **disorder.**

Study information for the parents of young participants

Project management: Prof. Dr. Simone Munsch, University of Fribourg, Switzerland

Dear Parents/Guardians

Your adolescent daughter/son is interested in participating in a clinical trial for the treatment of binge-eating disorder (BES; binge eating disorder) or regular binge eating. We would like to introduce the study to you below. [1]

More detailed information

1. Aim of the study

There are four studies in this project: "i-BEAT App", "i-BEAT VR", "i-BEAT Online" and "i-BEAT Epigenetics". With these we want to investigate different things:
- "i-BEAT App": Here we would like to learn more about the experience of social exclusion and its effects on mood and eating behaviour in adolescents and young adults with binge-eating disorder (BES: binge eating disorder) and/or regular loss of control, with the help of a smartphone app.
- "i-BEAT VR": In this laboratory study, we investigate the psychological and physiological effects of an interpersonal situation in virtual reality (VR).
- "i-BEAT Online": Here we are investigating the effectiveness of an internet and email-based treatment programme for the treatment of BES and/or regular loss of control eating in adolescents and young adults.
- "i-BEAT Epigenetics": Here we take a saliva sample through which we can examine how experiences in everyday life can affect the expression of genetic traits.

What all the studies have in common is that we want to use them to better understand how loss of control over eating occurs, what perpetuates this problem and how it can best be treated. While "i-BEAT App" focuses on the personal experience in everyday life, with "i-BEAT VR" and "i-BEAT Epigenetics" we are investigating even more closely how the experience of loss of control when eating is related to physical measures (e.g. the heartbeat) and genetics. With "i-BEAT online" we are trying to better understand how to optimally treat the issue.

2. Selection

All adolescents/young adults between the ages of 14 and 24 who suffer from binge eating disorder (BES: binge eating disorder), regular binge eating and/or a feeling of loss of control when eating, as well as people without these characteristics as a comparison group, can participate in this study. In addition, participants need a smartphone, internet access to be able to receive emails and a sufficiently good knowledge of German to be able to take part in one of the studies. Adolescents/young adults who also suffer from another serious mental illness that needs to be treated first or adolescents/young adults who are pregnant cannot participate. We will not conduct a pregnancy test, but will rely on the information provided by the participants. Also excluded from participation are adolescents and young adults who are in an employment relationship with Prof. Munsch or the working group of the Chair of Prof. Munsch or who work for Prof. Munsch.

[1] Your child is considered capable of judgement at 14 years of age or older and since the present study is classified as a minimal risk study, parental consent for participation is not legally required. However, we encourage the adolescents to inform their parents/guardians about the participation and the impressions gained.

3. General information

This study is conducted by the University of Fribourg, Switzerland and is led by Prof. Dr. Simone Munsch. With the help of the internet questionnaire that your child filled out, we were already able to gather initial information about his/her situation. Next, a more detailed interview will take place by telephone, which will last about an hour. Afterwards, your child can take part in one or more of the studies described. Participation in studies takes different lengths of time and takes place at different times.

"**i-BEAT App**" will last a total of 7 days. A total of 60 people will participate in this study. Here, the young people will be asked to report on his/her experiences and how he/she is feeling 3 times a day with the help of a smartphone app. This will take about 30 minutes each day. This study may start in the next few days. If your child participates in the "i-BEAT VR" study, we can use this opportunity to show him/her how to use the smartphone app. If your child does not participate in
"i-BEAT VR", we will instruct him/her about this by phone or e-mail.

"**i-BEAT VR**" will take place in the next few days at the University of Fribourg and will last about 2.5 hours. A total of 60 people will also participate in this study. Here, the young people will participate in a social situation or a game in virtual reality (VR). We will also measure his/her heartbeat and eye movements, which is not associated with any known side effects. In the course of this examination, we will also take a photo of the young people, which will be deleted again at the end of the examination.

"**i-BEAT Online**" will start in about 4 weeks and last about 15 weeks from then on. A total of 120 people will take part in this study. Here, the adolescents will participate in an internet-based therapy during the first 6 weeks of the study. After a subsequent 3-week break, your child will then take part in another 6-week therapy. One of the two therapies focuses on difficulties with eating, while the other therapy focuses on relationships with other people. It is randomly decided which of the two therapies will be started (i.e. both options are chosen with a probability of 50%). The treatment is based on a tested therapy programme for adults, which has now been adapted for adolescents/young adults. On the one hand, content is conveyed via the internet, while on the other hand, a weekly exchange with a trained therapist takes place via email.
Alternatively, it is possible to take up treatment at the psychotherapeutic practice centre of the University of Fribourg at regular conditions.

"i-BEAT **Epigenetics**" can take place as part of the "i-BEAT VR" study if your child is participating in it. We will then ask your child to take a swab from his/her cheek mucosa with a cotton swab. If your child does not take part in "i-BEAT VR", we will send the cotton swabs by post and ask that they be returned to us after the swab has been taken. A total of 60 people will also participate in this study. The investigation of these epigenetic processes does not allow any conclusions to be drawn about hereditary diseases, but we are investigating how environmental influences affect the hereditary dispositions, i.e. the reading of the genes.

In addition, we will ask the young people in about a year's time to complete the questionnaire they filled out at the beginning on the internet again. This way we can better understand how his/her situation has changed during this time.

We conduct this study in accordance with the laws in Switzerland. We also comply with all internationally recognised guidelines. The responsible cantonal ethics committee has reviewed and approved the study. A description of this study can also be found on the website of the Federal Office of Public Health: www.kofam.ch.

4. Procedure

After the adolescents have given their written consent to participate in the study, they will be given an appointment for a one-hour diagnostic interview over the phone. Through this interview and the questionnaires, we will check whether the adolescents meet the criteria for study participation and whether the treatment programme is suitable for them. If so, we will invite your child to participate voluntarily in one or more of the studies mentioned at the University of Fribourg at the Department of Psychology. [2]

The contact person in Fribourg who will accompany through the experimental investigations and take the buccal swabs is a student of clinical psychology and psychotherapy who will be trained by the study director. The treatment persons are trained psychologists at the Centre for Psychotherapy of the University of Fribourg

[2] Department of Psychology, University of Fribourg, Rue Faucigny 2, 1700 Fribourg.

and psychotherapists in advanced training who regularly consult with a psychotherapist certified by the Federal Office of Public Health (FOPH). The treatment persons are supported by Master's students from the Department of Clinical Psychology and Psychotherapy at the University of Fribourg. It will always be the same professional who accompanies them throughout the treatment.

The young people are free to discontinue their participation in the study at any time without any disadvantages for them. In rare cases, we may decide to exclude the young person from the study. This can happen if, in the course of the programme, we come to the conclusion that it would be better for the young person to take up another or additional treatment in order to be able to effectively reduce the burden. In this case, we would recommend a different or additional treatment offer. If the adolescents attend another treatment in addition to our programme, this is not necessarily a criterion for exclusion.

5. Benefit

The treatment programme in the i-BEAT Online study is based on a proven treatment approach adapted to adolescents/young adults. The other questionnaire studies and laboratory studies are designed to understand how the experience of social exclusion, mood and loss of control over eating are related. In this regard, no immediate benefit or harm is expected from participation.

6. Right

The young people participate in this study voluntarily. If they do not participate or later want to withdraw their participation, they do not have to justify this. Moreover, participation in the experimental studies is not a prerequisite for participation in the treatment programme. Psychological support is guaranteed regardless of this decision. The adolescents may ask questions about participation in the study at any time. To do so, they can contact the persons named at the end of this information. Since the interview and the questionnaire are used to check the criteria for participation in the study, we may have to exclude the adolescents from the study if they do not meet the participation criteria. If the interview indicates that there are other mental health problems in addition to binge eating disorder that require treatment, the adolescents will be informed of this and given information about treatment options, subject to their consent. The study staff are bound by the duty of confidentiality towards third parties, including you as parents/guardians. This means that without the consent of the young person, no information about the results of the interview and the course of the therapy will be passed on to you. Only in cases of acute danger to self (e.g. acute suicidal tendencies) or others will this confidentiality obligation be lifted and the young people as well as you as parents/guardians will be informed and included in the emergency procedure (see Risks and burdens for the participants).

7. Duties

As participants, it is necessary that the adolescents adhere to the guidelines and requirements of this study (including completing the sessions according to the schedule, regular contact with the treatment person). The implementation of the therapy programme requires that the adolescents are in a sober state (not under the influence of psychoactive substances such as drugs or alcohol) and that they can devote themselves to the content of the session in a quiet place during the email-based sessions. The young people undertake to report and inform about new symptoms, new complaints and changes in the way they feel if they are simultaneously receiving other treatments and therapies.

8. Risks and burdens for the participants

There is no risk for the adolescents by participating in this study and no undesirable side effects are to be expected. In the course of this treatment, the adolescents will intensively deal with themselves and various problem areas, which can be temporarily stressful. In addition, the young people are continuously accompanied by a treatment person. In acutely stressful situations, an emergency procedure is discussed and initiated. Information on crisis situations and behaviour as well as emergency contact details are handed out to the young people at the time of the psycho-diagnostic interview.
When entering the virtual world with VR goggles in one of the experimental examinations, so-called motion sickness may temporarily occur, the typical symptoms of which are dizziness or general malaise. If symptoms of motion sickness occur, we will immediately stop this examination in VR. The young people can also take off the VR glasses themselves at any time.
For adolescents/young adults of childbearing age: There are no risks and restrictions for adolescents/young adults of childbearing age. However, as pregnancy is associated with changes in eating, nutritional behaviour and body weight, these changes may affect the effectiveness of the programme. For this reason, we ask adolescents in pregnancy to postpone participation until 6 months after giving birth.

9. Other treatment options

Apart from the treatment offered and to be reviewed in the study, no other therapy option is offered in the study. However, it is possible to participate in individual psychotherapy for the treatment of binge-eating disorder (binge eating disorder) at the Psychotherapeutic Practice Centre of the University of Fribourg (https://www3.unifr.ch/psycho/de/psychotherapie) instead of participating in the present treatment study (subject to a fee).

10. Results from the study

The study management will inform the adolescents during the study of any new findings that may affect the benefit of the study or its safety and thus their consent to participate in the study. The adolescents will receive this information verbally and in writing.

11. Confidentiality of data and samples

Personal, psychological and medical data will be collected for this study. Very few professionals will see the unencoded data, and this is solely to fulfil tasks within the study. When data is collected for study purposes, it will be encrypted (coded). Encryption means that all personal data that could identify a young person (name, date of birth) are deleted and replaced by a key/code. The key list always remains in the institution (University of Fribourg). Those persons who do not know the key can therefore not draw any conclusions about a person. If the results of the study are published, they do not reveal any information about an individual person. Names never appear on the internet or in a publication. The individual data (so-called raw data) are made publicly available on a digital database so that they can be used for future research purposes. However, this data is always encrypted and can never be traced back to an individual. All persons who have access to the data within the framework of the study are subject to the duty of confidentiality. Data protection regulations are adhered to and the young people have the right to access their data at any time.
The buccal swabs are sent in encrypted form to a database/biobank in Bochum, Germany, where they are examined for this project and then immediately destroyed. The key list remains in the institution (University of Fribourg) and only the study management and the treatment staff have access to it. The study management is responsible for ensuring that the same standards are observed abroad as in Switzerland. The competent ethics committee may be given access to the data in order to check data security. The data may also be reviewed by the institution that initiated the study. The study director may have to disclose personal data for such checks. All persons must maintain absolute confidentiality.

12. Resignation

The young people can stop and withdraw from the study at any time if they wish. The data and samples collected up to that point will continue to be evaluated in encrypted form, since information from people who do not want to or cannot complete the treatment is just as important for improving the treatment offer. After the evaluation, the data will be completely encrypted, i.e. the key allocation will be destroyed so that afterwards no one can find out who the data originally came from.

13. Compensation for participants

If you are not studying psychology at the University of Fribourg/Freiburg, your effort will be compensated in the following way. For participation in the "i-BEAT App" study, the young people will receive 100 CHF, and for participation in the "i-BEAT VR" study, including participation in the "i-BEAT Epigenetics" study, they will be compensated 150 CHF. If the adolescents are studying psychology at the University of Fribourg/Freiburg, they will receive subject hours for their participation in the studies "i-BEAT App", "i-BEAT VR" and "i-BEAT Epigenetics". Please take into account that students of psychology can compensate required experimental subject hours by substitute performances (see Bachelor plan, page 17, item 9 : Experimental self-awareness www3.unifr.ch/psycho/en/assets/public/bachelor/plans/PLE_Psycho_BA180_De_rat29012019v03.pdf). Participation in the treatment programme "i-BEAT Online" is free of charge. Expenses such as travel expenses, which are only due to participation, are additionally reimbursed. Participation in the study does not entitle the young people to any commercial developments (e.g. patents).

14. Liability

The University of Fribourg and thus the project management by Prof. Simone Munsch, who initiated the study and is responsible for its implementation, is liable for any damage that might be caused to the young people in connection with the research. The requirements and the procedure for this are regulated by law. For this purpose, the University of Fribourg has taken out an insurance policy with the Basler Versicherungen in order to be able to cover liability in the event of a possible claim during the implementation of the experimental studies or the treatment programme. If the young people suffer damage, they are asked to contact the study director, Prof. Simone Munsch.

15. Funding of the study

The study is funded by the Swiss National Science Foundation (SNF 10001C_185387 / 1).

16. Contact persons

In case of questions, uncertainties or emergencies arising during the study or afterwards, the young people and you can contact one of these contact persons at any time.

Prof. Dr. Simone Munsch
Chair of Clinical Psychology and Psychotherapy
Department of Psychology
University of Fribourg
Rue de Faucigny 2
CH-1700 Fribourg
Telephone Prof. Dr. Simone Munsch: + 41 26 300 76 57
Email: simone.munsch@unifr.ch

M.Sc. Felicitas Forrer
Graduate assistant
RM 01 bu. C-2.118
Rue P.A. de Faucigny 2
CH-1700 Fribourg
Tel. +41 26 300 7658
Email: felicitas.forrer@unifr.ch

M.Sc. Verena Müller
SNF doctoral student
RM 01 bu. C-2.118
Rue P.A. de Faucigny 2
CH-1700 Fribourg
Tel. M.Sc. Verena Müller: +41 26 300 7659
Email: verena.mueller@unifr.ch

M.Sc. Adrian Naas
SNSF doctoral student
RM 01 bu. C-2.113
Rue P.A. de Faucigny 2
CH-1700 Fribourg
Tel. M.Sc. Adrian Naas: +41 26 300 7478
Email: adrian.naas@unifr.ch

In psychological emergency situations, you can also dial the 24-hour emergency number of the Swiss worry line (Dargebotene Hand): 143.

17. Glossary (terms requiring explanation):

Binge eating disorder: According to diagnostic criteria described in the Diagnostic and Statistical Manual of Mental Disorders (DSM-5, APA, 2013), this form of eating disorder is characterised by eating a large amount of food in a definable period of time and losing control over what and how much they eat. These so-called "eating attacks" are often accompanied by eating very quickly, in a jumbled manner, without hunger and to the

point of an unpleasant feeling of fullness. As a result of these eating binges, feelings of shame, guilt and dejection set in.

Epigenetics: Field of biology and medicine that deals with which gene becomes active under which environmental influences. Epigenetic findings can, for example, help to understand under which biological and environmental conditions adolescents/young adults are more prone to binge eating.

University of Fribourg Switzerland
Department of Psychology
Prof. Dr. Simone Munsch
UNI FR
UNIVERSITÉ DE FRIBOURG
UNIVERSITÄT FREIBURG

i-BEAT
Binge Eating Adolescent And Young Adults Treatment
Ein Forschungs- und Internet-basiertes Behandlungsprogramm für Jugendliche und junge Erwachsene mit Essanfällen
Universität Freiburg, Schweiz ► i-BEAT@unifr.ch

D7

Short title: i-BEAT - Binge-Eating Adolescent And Young Adults Treatment - a research and internet-based treatment programme for adolescents and young adults with binge eating disorders

Consent form for audio recording of the Diagnostic Interview in Mental Disorders (DIPS; Margraf et al., 2017)

Project management: Prof. Dr. Simone Munsch, University of Fribourg, Switzerland

Dear participant,

You have indicated elsewhere that you are interested in participating in one of the i-BEAT sub-studies "i-BEAT VR" or "i-BEAT Online". Thank you very much for this. Prior to these two sub-studies, the so-called Diagnostic Interview in Mental Disorders (DIPS) will be conducted. This interview will be conducted by MS teams and as part of the process the interview will be recorded (audio recording). This consent form supplements the previously signed consent form(s) for participation in one or more other i-BEAT sub-study(s).

1. Aim of the DIPS

With the DIPS we carry out a comprehensive psychological diagnosis, which gives us an impression of your general mental health. We can also use the DIPS to clarify whether you suffer from a serious mental disorder that should be treated before your participation in the study.

2. Procedure of the DIPS

After you have given your written consent to participate in the "i-BEAT VR" or "i-BEAT Online" sub-study, you will receive an appointment for your DIPS. The DIPS will take place via MS Teams. We will send you further information about the appointment by e-mail. During the diagnostic interview, you will be asked various questions about possible mental health problems.

3. Recording of the DIPS

As part of this study, an audio recording of the DIPS interview will be made in order to assess the comparability of the diagnostic interviews. Your information and data will be treated confidentially.

4. Data confidentiality

The recordings of the diagnostic interviews are encrypted and stored on a server at the University of Fribourg in Switzerland. Only the study director (Prof. Simone Munsch), the computer scientist of the Psychology Department of UNIFR: Thierry Progin, who is also bound by confidentiality, and the study collaborator M.Sc. Adrian Naas know the key to the stored recording. Without this key, the soundtrack on the server will remain distorted. To improve the security of your personal data, all persons conducting diagnostic interviews at i-BEAT sign a confidentiality agreement. This confirms that they guarantee that no one will listen or be present when they conduct the DIPS and that they will delete the interview from the computer once it has been saved on the server.

5. Right

Your participation in the study and in DIPS is voluntary and you can end it at any time without giving reasons. The study staff are subject to a duty of confidentiality towards third parties. This means that no information about your data will be passed on without your consent. Your collected data will be treated confidentially (see point 4).

6. Contact person(s)

If you have any questions or uncertainties that arise during or after the survey, you can always contact one of these contact persons:

PI Prof. Dr. Simone Munsch
Chair of Clinical Psychology and Psychotherapy
Department of Psychology
University of Fribourg
Rue de Faucigny 2
CH-1700 Fribourg
Tel. Prof. Dr. Simone Munsch: + 41 26 300 76 55
Email: simone.munsch@unifr.ch

M.Sc. Felicitas Forrer
Graduate assistant
RM 01 bu. C-2.118
Rue P.A. de Faucigny 2
CH-1700 Fribourg
Tel. +41 26 300 7658
Email: felicitas.forrer@unifr.ch

M.Sc. Verena Müller
SNF doctoral student
RM 01 bu. C-2.118
Rue P.A. de Faucigny 2
CH-1700 Fribourg
Phone M.Sc. Verena Müller: +41 26 300 7659
Email: verena.mueller@unifr.ch

M.Sc. Adrian Naas
SNSF doctoral student
RM 01 bu. C-2.113
Rue P.A. de Faucigny 2
CH-1700 Fribourg
Phone M.Sc. Adrian Naas: +41 26 300 7478
Email: adrian.naas@unifr.ch

With my signature I expressly declare that...
(please tick)

- ❒ **my questions in** connection with the Diagnostic Interview for Mental Disorders (DIPS) and its recording have been **answered**. I will receive a copy of my written informed consent.
- ❒ I **have been informed of** this in writing **and that** I **consent to the audio track of my DIPS being recorded and stored in encrypted form.**
- ❒ I don't want the DIPS to be recorded on audio.

Place, Date, Signature Participant

____

UNIVERSITY OF FRIBOURG SWITZERLAND
DEPARTMENT OF PSYCHOLOGY
PROF. DR. SIMONE MUNSCH

UNI
FR
UNIVERSITÉ DE FRIBOURG
UNIVERSITÄT FREIBURG

D8

PROF. DR. SIMONE MUNSCH
Psychotherapeutic practice centre
Clinical Psychology and Psychotherapy
Rue de Faucigny 2,
CH-1700 Fribourg (Switzerland)
Tel.+41 (0)26 300 76 57
E-mail: simone.munsch@unifr.ch

Declaration of confidentiality for the implementation of DIPS

A Diagnostic Interview for the Assessment of Mental Disorders (DIPS) must always be conducted in the trainee's office or in the garden office. In the following exceptional situations, it is possible to conduct the interview at home: At off-peak times, e.g. at the weekend or in the evening.

This consent form serves to clarify the conditions for conducting DIPS in the home office. It requires that a declaration of confidentiality signed by the person conducting the interview is available.

The following are some of the conditions to be able to conduct a DIPS in the home office:

- The session takes place in a quiet room with the use of headphones.
- It must be ensured that no person in the vicinity can listen in (e.g. roommate).
- In order to record only an audio track, they turn off the camera after the greeting and ask the adolescents or young adults to do the same (no image recording).
- The interviews are recorded. Project staff ensure that the audio stream is downloaded locally immediately after the recording and stored exclusively on the UNIFR server located in Switzerland in password-protected and access-restricted folders. Afterwards, the audio recordings are immediately deleted wherever MS Teams stores the recordings.
- To initiate the upload to the secured UNIFR server, save the audio track on a local storage medium (USB stick, SD card or hard drive) and hand over this single copy of the recording to M.Sc. Adrian Naas.

I undertake to act in accordance with the information on this consent form. I expressly declare that I understand the information and have no further questions.

Place, date, signature

Place, date, signature

________________________________ ________________________________

Prof. Dr. Simone Munsch
Clinical psychology and psychotherapy
PI i-BEAT

Biological specimens	33	Plans for collection, laboratory evaluation, and storage of biological specimens for genetic or molecular analysis in the current trial and for future use in ancillary studies, if applicable

The below mentioned rational and procedure has been evaluated by the local SNSF for scientistic quality and approved by the ethical committee of the canton of Berne

Background
We explore biological underpinnings of the susceptibility to rejection sensitivity and related phenomenon and investigate epigenetic factors influencing the activation of the threat- related biological systems and emotion regulation capacity (Hellhammer, Wust, & Kudielka, 2009; Lam, Dickerson, Zoccola, & Zaldivar, 2009). There has been evidence that life- adversities may influence DNA methylation patterns across various tissues such as brain, saliva and blood samples (Barker, Walton, & Cecil, 2018; Kumsta et al., 2016). A recent review by Barker on effects of in-family adverse experiences summarized that specific types of stress such as physical abuse or neglect impact differently on systems and it was stated that victimization outside of the family represents a still under researched domain (Barker et al., 2018). In relation with the role of rejection sensitivity in LOC, DNA methylation of the FK-506- binding protein 5 *(FKBP5)* is of interest, as FKBP5 is a critical regulator of the HPA system through effects on glucocorticoid receptor sensitivity (Lester et al., 2016; Roberts et al., 2015). In addition, the DNA methylation of the glucocorticoid receptor gene (*NR3C1*) seems to be related to the activation of the hypothalamic-pituitary adrenocortical (HPA) axis in children of distressed mothers (Oberlander et al., 2008). Another important neuropeptide hormone, oxytocin has been implicated in coping with social stress (Auer, Byrd-Craven, Grant, & Granger, 2015) and emotion regulation in mental disorders (Auer et al., 2015; Unternaehrer et al., 2012) and plays an essential role in appetite regulation. A more pronounced methylation of the oxytocin receptor gene (*OXTR*) was associated with bulimic behavior and the activation of avoidance behaviors in women with BN compared to AN or healthy women (Kim, Kim, Kim, Shin, & Treasure, 2015). Furthermore, recently the SLC6A4 transmembrane serotonin transporter gene has been linked to eating behaviour (e.g. Lillycrop et al., 2019), with stress reactivity (Alexander et al., 2014) and most importantly to treatment response (Domschke et al., 2014). We will further explore interpretable epigenetic underpinnings DNA methylation of predictors of effects and changes prior and after treatment (Mill & Heijmans, 2013). The current evidence of epigenetic changes after psychotherapy is limited and there is no study which investigated epigenetic effects in patients with LOC. To date there are 6 studies in children and adults investigating the effect of CBT or DBT in anxiety and borderline personality disorder on DNA methylation change and of the predictive value of DNA methylation of candidate genes on treatment outcome (Eley, 2014; Kumsta, submitted). A commonality of these studies was that change in DNA methylation was associated with treatment response, with responders and non-responders showing divergent patterns of DNA methylation change. Pretreatment DNA methylation was not associated with therapy outcome in most cases. Among this preliminary research, a study with 10 years old children with anxiety disorders found no association between treatment response and pre-treatment methylation of the FK506-binding protein 5 *(FKBP5)* gene measured in children's saliva but an interaction of methylation change with response to CBT (changes in behavior, emotional coping and cognitive style) (Lester et al., 2016; Roberts et al., 2015). Existing data suggests that even though peripheral cells such as buccal epithelium do not reflect DNA methylation status of brain tissue, they offer a valuable peripheral epigenetic marker for treatment outcome in different age groups and gender. The present study therefore explores DNA methylation levels of genes involved in stress response (*glucocorticoid receptor gene: NR3C1,*

FK506-binding protein 5: FKBP5, transmembrane serotonin transporter gene: *SLC6A4*) as well as in saturation, emotion regulation and social affiliation *(oxytocin receptor gene: OXTR)* in youth with LOC prior and after treatment compared to a healthy control group. Specifically, we will test whether DNA methylation levels differentiate between a group of healthy youth and youth with LOC, whether DNA methylation levels predict treatment outcome, whether they change in response to treatment, and whether treatment response is associated with a direction of change.

Exploratory aims

- percentage of DNA methylation of FK506-binding protein 5 (*FKBP5)*, glucocorticoid receptor gene (*NR3C1),* transmembrane serotonin transporter gene (*SLC6A4*) and oxytocin receptor gene (*OXTR)* between youth with LOC eating (prior to treatment) and a HCG
- Predictive value and change of DNA methylation of FK506-binding protein 5 (*FKBP5)*, glucocorticoid receptor gene *(NR3C1),* transmembrane serotonin transporter gene (*SLC6A4*) and oxytocin receptor gene (*OXTR)* in youth with LOC eating (prior and after i-BEAT)

Assessment, storage, data security.

Epigenetic markers will be assessed before and after treatment either in the lab when participating in substudy 2 or at home, in order to investigate the association with rejection sensitivity and to evaluate their association compared to patterns in a HCG and to evaluate changes in DNA methylation during the treatment. Participants will be instructed to brush the inside of their cheeks with standard DNA collection devices (Analytik Jena). DNA brushes will be coded and stored at -30°C in lockable freezers at the Department of Psychology of Fribourg.

Epigenetic data in this study is not identified by participant name but by a unique participant number. Biological material is appropriately stored in a restricted area only accessible to the authorized personnel. Coding/ encryption is applied to all biological data transmitted to our collaborator Prof. Robert Kumsta from the Ruhr-University of Bochum, a specialist lab regarding epigenetics and DNA analysis. At no point will Prof Kumsta and his team be able to decipher personal information of the genetic material that is being sent to them.

Data will be assessed and coded, so that the participant cannot be identified by any other person than the sponsor and the study organization (M.Sc. Felicitas Forrer, M.Sc. Verena Müller, M.Sc. Adrian Naas). The list with code and names is kept under lock in the administrative office of the sponsor.

Analysis

Upon study completion, the samples will be shipped to the collaborator’s affiliation at the Department of Genetic Psychology, Ruhr-University Bochum, Germany to be analyzed (see consumables, project partner Prof. Robert Kumsta). Genomic DNA will be extracted from buccal epithlium sampled with the blackPREP Swab DNA Kit (Analytik Jena). Sodium bisulfite conversion of 300ng genomic DNA will be performed using Zymo EZ DNA Methylation-Gold Kit following the manufacturers’ standard protocol. DNA methylation of selected candidate genes will be quantified using targeted next generation sequencing on the Illumina MiSeq platform. The Department of Genetic Psychology is equipped with all necessary instrumentation. Isolated DNA will be stored in locked freezers at the Department of Genetic Psychology in Bochum and will be destroyed after study completion.

All data of the i-BEAT study is subjected to open access.
